# Comparative and Functional Genomics of *Rhodococcus opacus* PD630 for Biofuels Development

**DOI:** 10.1371/journal.pgen.1002219

**Published:** 2011-09-08

**Authors:** Jason W. Holder, Jil C. Ulrich, Anthony C. DeBono, Paul A. Godfrey, Christopher A. Desjardins, Jeremy Zucker, Qiandong Zeng, Alex L. B. Leach, Ion Ghiviriga, Christine Dancel, Thomas Abeel, Dirk Gevers, Chinnappa D. Kodira, Brian Desany, Jason P. Affourtit, Bruce W. Birren, Anthony J. Sinskey

**Affiliations:** 1 Department of Biology, Massachusetts Institute of Technology, Cambridge, Massachusetts, United States of America; 2 The Broad Institute, Cambridge, Massachusetts, United States of America; 3 Department of Chemistry, University of Florida, Gainesville, Florida, United States of America; 4 454 Life Sciences, Branford, Connecticut, United States of America; 5 Division of Health Sciences and Technology, Massachusetts Institute of Technology, Cambridge, Massachusetts, United States of America; 6 Engineering Systems Division, Massachusetts Institute of Technology, Cambridge, Massachusetts, United States of America; Progentech, United States of America

## Abstract

The Actinomycetales bacteria *Rhodococcus opacus* PD630 and *Rhodococcus jostii* RHA1 bioconvert a diverse range of organic substrates through lipid biosynthesis into large quantities of energy-rich triacylglycerols (TAGs). To describe the genetic basis of the *Rhodococcus* oleaginous metabolism, we sequenced and performed comparative analysis of the 9.27 Mb *R. opacus* PD630 genome. Metabolic-reconstruction assigned 2017 enzymatic reactions to the 8632 *R. opacus* PD630 genes we identified. Of these, 261 genes were implicated in the *R. opacus* PD630 TAGs cycle by metabolic reconstruction and gene family analysis. *Rhodococcus* synthesizes uncommon straight-chain odd-carbon fatty acids in high abundance and stores them as TAGs. We have identified these to be pentadecanoic, heptadecanoic, and cis-heptadecenoic acids. To identify bioconversion pathways, we screened *R. opacus* PD630, *R. jostii* RHA1, *Ralstonia eutropha* H16, and *C. glutamicum* 13032 for growth on 190 compounds. The results of the catabolic screen, phylogenetic analysis of the TAGs cycle enzymes, and metabolic product characterizations were integrated into a working model of prokaryotic oleaginy.

## Author Summary

Biofuels research is focused on understanding the energy-related metabolic capabilities of a broad range of biological species. To this end we sequenced the genome of *Rhodococcus opacus* PD630, a bacterium that accumulates close to 80% of its cellular dry weight in oil, a rare trait in the prokaryotic and eukaryotic kingdoms. *R. opacus* PD630 has a large 9.27 Mb genome that contains many homologous genes dedicated to lipid metabolism. The number and novelty of these predicted genes presents a challenge to the complete and accurate metabolic reconstruction of this species' metabolism based only on genome sequence. To refine our sequence-based metabolic reconstruction, we developed a multidisciplinary approach that included integrating the identification of abundant yet uncommon straight-chain odd-carbon lipid biosynthesis and the results of a catabolic screen for growth substrates. Comparative analysis of the *R. opacus* PD630 genome sequence with those of a group of related species provided a view into how this bacterium became such a remarkable TAGs producer and led to the identification of a set of biofuels target genes for this group of bacteria. Our synthesis of genome sequence and phenotypic information supports a model for the genetic basis for prokaryotic oleaginy and provides key insights for the engineering of next-generation biofuels with genes that are conserved in both prokaryotic and eukaryotic kingdoms.

## Introduction

Bio-Diesel is an energy-rich portable fuel derived mainly from triacylglycerols (TAGs). Biodiesel and related fuels are extracted from oleaginous organisms, both photosynthetic and non-photosynthetic, that use available energy sources to fix carbon into high levels of stored lipids. In chemoheterotrophic organisms TAGs are synthesized by bioconversion of organic compounds such as the sugars and organic acids derived from globally-abundant cellulosic biomass. A genetic understanding of the oleaginous metabolism of chemoheterotrophic species like *Rhodococcus* provides critical insight for biofuels development.

The high GC content Gram-positive Actinomycetales bacteria *Rhodococcus opacus* PD630 and *Rhodococcus jostii* RHA1, a close relative that has a completely sequenced genome [1], were previously shown to accumulate large amounts of TAGs and wax esters (WEs) [2], [3], [4]. *Rhodococcus* species present an attractive target for industrial processes due to high substrate tolerances and high density culturing on a rapid time scale as compared to many photosynthetic organisms [5], [6]. The oleaginous metabolism of *Rhodococcus* goes beyond abundant lipid biosynthesis to include diverse hydrocarbon catabolism. *R. jostii* RHA1 was isolated from soil containing 1,2,3,4,5,6-hexachlorocyclohexane (Lindane) [7], while *R. opacus* PD630 was enriched on phenyldecane as a sole carbon source after isolation from soil sampled at a gas works plant [2]. *Rhodococcus* can catabolize and detoxify several aromatic hydrocarbons that contaminate soil from industrial waste products. These toxic substrates include polychlorinated biphenyls (PCBs) [8], [9], [10], [11] and other halogenated compounds such as Lindane that was used in large quantity for agricultural practices.

Limitation of an essential nutrient stimulates enzymatic conversion of the non-limiting essential nutrients into stored polymers such as phosphorous conversion to poly-phosphate [4], acetyl- and other short acyl-CoAs conversion to polyhydroxyalkanoates (PHAs) [12], [13], [14], or the production of TAGs and WEs from these same short chain acyl-CoA primers [2], [15]. Most prokaryotes store carbon as polyhydroxyalkanoic acids (PHAs) when other essential nutrients such as reduced nitrogen are limiting. By contrast bacteria in the order Actinomycetales have uniquely developed a storage lipid cycle that leads to accumulation of TAGs and WEs [16]. Abundant TAGs accumulation in *Rhodococcus* provides a pool of fatty acids for β-oxidation as cellular fuel, components of the plasma membrane, and substrates for the enzymatic production of the very-long and highly-modified extracellular lipids characteristic of Actinomycetales.

Lipid metabolism in the genus *Mycobacterium* has been a major focus of scientific research due to the effect pharmacological inhibitors of lipid biosynthesis such as isoniazid [17], [18], [19], thiolactamycin, and pyrazinamide [20] have on killing pathogenic mycobacteria. Whole-genome views of lipid metabolism in mycobacteria reveal these bacteria have developed several lipid biosynthesis systems and a large number of genes to support diverse and abundant lipid biosynthesis. In *Mycobacterium* an order to the enzyme activity of the lipid synthases has been established through genetic, biochemical, and pharmacological evidence; wherein lipids are biosynthesized *de novo* by the multifunctional FAS type 1a enzyme followed by further elongation via the FAS II system and the multifunctional MAS-family type 1b synthases. Collectively these 3 fatty acid synthase systems produce 2 classes of fatty acyl-CoAs that differ in chain length. One class contains lipids <20 carbons (C_20_) that are components of the plasma membrane and the storage lipids TAGs and WEs. Another class contains lipids >C_26_ that are built by multiple synthases and can reach lengths as long as C_90_ in some *Mycobacterium* species [19], [21] but only C_60_ in *Rhodococcus opacus* [22] and C_54_ molecules have been observed in *R. equi* [23]. The longer chain length lipids are used in Actinomycetales to build a protective extracellular coat that helps these bacteria survive in harsh environments whether it be the phagosome of a macrophage or contaminated soil enriched in toxic organic compounds. The interplay between multiple lipid biosynthesis systems in Actinomycetales [19] requires genetic understanding for engineering the flow of carbon to desired lipid types.

## Results

### Metabolic Reconstruction of *Rhodococcus opacus* PD630

To establish a genetic model of *Rhodococcus* metabolism, we generated a high quality draft sequence of the *Rhodococcus opacus* PD630 genome. DNA sequencing with 454 shotgun and 3 kb paired-end reads resulted in 16 large scaffolds containing 9.27 Mb of assembled DNA sequence. We stitched the gapped genome scaffolds based on extensive chromosomal-synteny with the complete genome sequences of related *Rhodococcus* species *R. jostii* RHA1 and *R. opacus* B4 (Figure S1).

The *R. opacus* PD630 genome contained 8632 genes that underwent metabolic reconstruction using pathway tools software [24] resulting in a model containing 1735 metabolic reactions. Enzymes were connected to metabolic reactions based on enzyme commission numbers (EC#) that were assigned by the EFICAz2 algorithm [25] and by gene-name recognition within pathway tools software. This automated EC# assignment allowed for multiple genomes to undergo metabolic reconstruction in parallel. Comparisons between metabolic reconstructions for a set of 8 phylogenetically related and one outlier species *Ralstonia eutropha* H16 that were assembled in this way can be browsed at (http://tinyurl.com/opacuscyc14-5-comparative). The resulting initial metabolic reconstruction of *R. opacus* PD630 Opacuscyc14.5_comparative contains 400 metabolic pathways and 135 transport reactions.

A more complete metabolic reconstruction of *R. opacus* PD630 by pathway hole filling using the pathway tools 14.5 software, additional EC # assignments made with the database at Kyoto Encyclopedia of Gene and Genomes KEGG (http://www.genome.jp/kegg/), and limited manual curation (outlined in Figure S2) resulted in a metabolic reconstruction containing an additional 282 metabolic reactions, 44 metabolic pathways, and eight transport reactions. Opacuscyc_14.5 was improved by refining the metabolic model of TAGs biosynthesis and degradation by metabolic product characterization of uncommon fatty acids that accumulate to high levels in *Rhodococcus*. The results of a screen for bacterial growth on 190 metabolic compounds were used as a multi-genic test of the reconstruction, described in more detail below. Comparison of the results of the catabolic screen with the metabolic pathway predictions revealed that precision was 65% before refinement and 71% after refinement (Table S1). The current working model of Opacuscyc14.5 contains 2017 metabolic reactions, 444 metabolic pathways, and 143 transport reactions that can be browsed at (http://tinyurl.com/4dv5m32).

### *Rhodococcus* Oleaginy Resulted from Key Genes That Emerged in Actinomycetales

**Figure 1:**
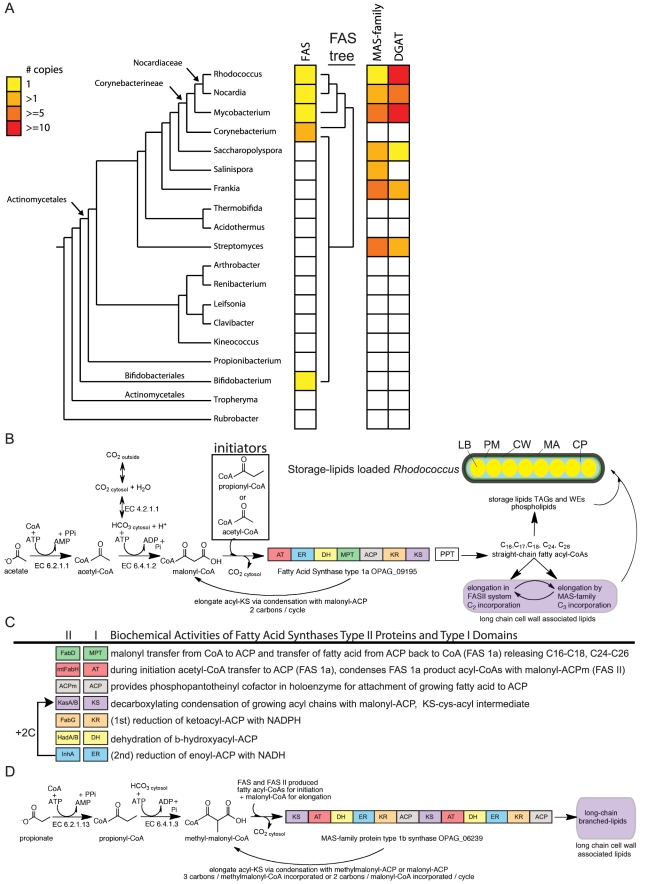
Phylogenetic and metabolic pathway features of lipid biosynthesis in *Rhodococcus*. a) A phylogenetic representation of oleaginous genes in Actinomycetales. An AMPHORA-based tree of related genera (left) provides context to the copy number of unique oleaginous genes within each genera presented in three columns to the right. Remarkable oleaginous genes in Actinobacteria are fatty acid synthase (FAS) type 1a, MAS-family type 1b, and diacylglycerol acyl-transferases (DGATs). Oleaginous gene family members in each genus were counted and color-coded from 0 to >10. A phylogenetic tree of the FAS type 1a gene is presented to the right of the FAS column. b) Fatty acids biosynthesis in *Rhodococcus*. Acetyl-CoA is the product of many biochemical reactions and a limiting substrate in lipid biosynthesis. Acetyl-CoA can also be generated from acetic acid found in the environment by ligation to –CoA. ATP-hydrolysis enables bicarbonate coupling to acetyl-CoA forming malonyl-CoA, the substrate used for elongation. The lipid biosynthesis -CoA substrates are incorporated in *Rhodococcus* by three fatty acid biosynthesis systems that begins with multifunctional FAS type 1a. This synthase contains the following enzyme activities: malonyl palmityl transferase (MPT), acetyl transferase (AT), keto synthase (KS), keto reductase (KR), dehydratase (DH), enoyl reductase (ER), acyl carrier protein (ACP) catalyzing decarboxylating condensation of malonyl-ACP with acetyl-CoA, propionoyl-CoA, or acyl-CoAs to generate straight-chain fatty acyl-CoAs that range in size (C_16_–C_26_) and include odd-carbon fatty acids. Phosphopantotheinyl transferase (PPT) converts the ACP domain of FAS into the active form via attachment of phosphopantotheinate to a serine residue and is encoded by the down stream gene in the *Rhodococcus* bicistronic FAS operon. During inititiation malonyl-ACP is condensed with either acetyl-CoA or propionoyl-CoA to form a C_4_ or C_5_ straight-chain intermediate, respectively. Further elongation cycles consume malonyl-ACP for C_2_ additions with concomitant release of CO_2_ for each round of Claisen-type condensation reaction. The fatty acyl-CoA products of FAS <C_20_ are attached to glycerol catalyzing production of phospholipids and triacylglycerols (TAGs) or processed by number of other routes. Fatty acyl-CoAs can be further elongated by the FAS II system or MAS-family proteins. Wax esters (WEs) are generated by transesterification of fatty acyl-CoAs with fatty alcohols. TAGs, WEs, phospholipds, FAS II and MAS products come together to form compartments within *Rhodococcus*. Lipid bodies (yellow, LB) house the stored lipids surrounded by cytoplasm (blue, CP). The Plasma Membrane (orange, PM) is surrounded by cell wall (green, CW). The mycolic acid layer (black, MA) on the outside of *Rhodococcus* cells contains the long chain lipids. 1c) Comparison of biochemical activities of type II proteins and type I protein domains. Colors of FAS II genes correspond to related function in type 1 protein domains. Notable differences between synthase type 1 domains and type 2 proteins include: the MPT domain is dual functional in FAS type 1a thus releasing –CoA products through palmitoyl-CoA activity. The AT domain in type 1a provides acetyl-CoA and propionoyl-CoA substrates for condensation. In type 1b synthase the AT domain provides long chain acyl-CoAs for condensation on the KS domains. 1d) Methyl-branched lipid biosynthesis model for type 1b synthase activity from related mycobacterial protein PKS12. The C_3_ organic salt propionate can be ligated to CoA generating propionoyl-CoA, a substrate for *Rhodococus* lipid biosynthesis that is also produced in several metabolic degradation pathways. Propionoyl-CoA can be used directly by FAS during initiation or carboxylated to form methylmalonyl-CoA making it a substrate for a second multifunctional fatty acid synthase with a type 1b protein domain architecture present in *Rhodococcus* OPAG_06239. The type 1b synthase belongs to the mycocerosic acid synthase (MAS)-family of proteins that in *Mycobacterium* were shown to incorporate methylmalonyl-CoA into growing fatty acid chains creating methyl-branched lipids.

**Figure 2:**
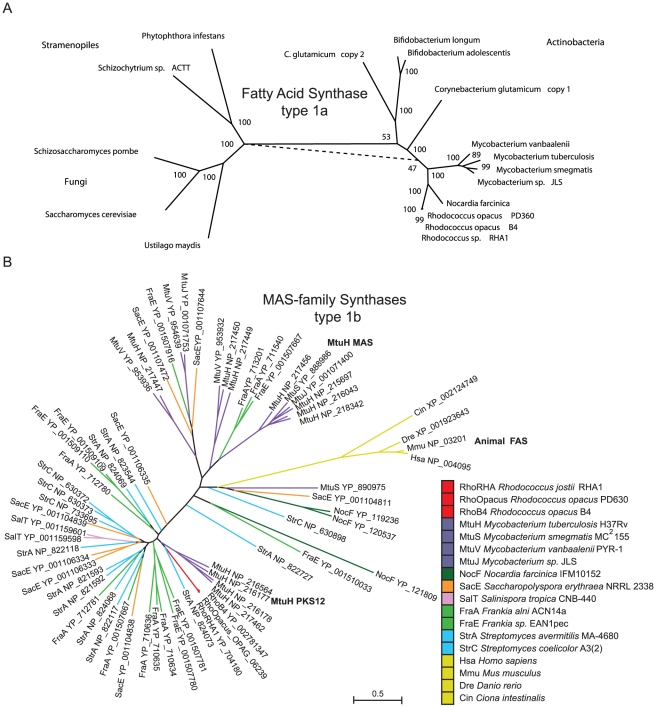
Phylogenetic analysis of type 1a and type 1b fatty acids synthases. a) A phylogenetic tree that shows FAS type 1a is found in Actinobacteria, Stramenopiles, and Fungi. b) A phylogenetic tree of MAS-family type 1b proteins. This gene family is expanded in the genera *Mycobacterium*, *Frankia*, and *Streptomyces* but present in single copy in *Rhodococcus* belonging to branch containing the PKS12 from *M. tuberculosis* NP_216564 that also contains similar protein domain architecture.

Several eubacteria of the order of Actinobacteria including *Rhodococcus*, *Corynebacterium*, *Mycobacterium*, and *Bifidobacterium* are distinguished for having both the type 1a fatty acid synthase (FAS) (Figure 1a) and a FAS II lipid biosynthesis system. FAS is a polyketide synthase related protein containing all of the necessary enzymatic activities for *de novo* lipid biosynthesis (Figure 1b). In mycobacteria FAS has been shown to elongate C_2_ and C_3_ carbon acyl-CoAs into C_16_, C_18_, C_24_, and C_26_ fatty acyl-CoAs [26], [27]. In the suborder of bacteria Corynebacterineae, the FAS II system of lipid biosynthesis elongates FAS product fatty acyl-CoAs through the enzymatic activity of 6 families of enzymes (Figure 1c) for biosynthesis of mycolic acids and related cell wall associated lipids. The FAS II system in these Actinomycetales operates on C_16_–C_26_ length fatty acyl-CoAs not the usual short chain lipid biosynthesis substrates characteristic of the related enzymes in bacteria, chloroplasts, and mitochondria. The number of unique FAS genes is shown in Figure 1a in the context of a phylogenetic tree of genera built by AMPHORA [28]. Unlike the widespread taxonomic representation of the FAS II genes, the FAS type 1a gene in *Rhodococcus* has genus representation in prokaryotes is limited to only within Actinomycetales. The FAS gene is likely to have emerged in Actinobacteria (Figure 1a) and was horizontally transferred to only those eukaryotic branches containing fungi and stramenopiles (Figure 2a).

Our genome-based metabolic reconstruction revealed close metabolic pathway relationships when we compared the lipid metabolism enzymes of the phylogenetically related genera *Mycobacterium*, *Nocardia*, and *Rhodococcus*. These Actinomycetales contain multiple pathways for lipid biosynthesis utilizing a unique combination of multifunctional fatty acid synthases (FAS), a type 1a synthase and a related MAS-family type 1b synthase that generate linear- and branched-fatty acids respectively (Figure 1D). Diacyl glycerol acyl transferase (DGAT) enzymes convert fatty and other carboxylic acids into TAGs [29]. DGAT genes are also of limited genus representation in eubacteria and interestingly are present in six out of seven genera that also contain at least one gene of the MAS-family type 1b synthases (Figure 1a). Closely related genera to *Rhodococcus* share expanded gene-families of DGATs [29], [30]. A phylogenetic tree based on 701 shared genes present in a single copy and general genomic features of representative species used for comparisons with *Rhodococcus* are presented in Figure S3.

The phylogenetic distance between the bacterial taxa that contain the FAS type 1a suggest this gene has likely been horizontally transferred within Actinomycetales. The FAS gene was duplicated in *Corynebacterium* and horizontally transferred between *Bifidobacterium* and *Corynebacterium*. The FAS type 1a protein from *Rhodococcus* is highly related, ranging between 60–65% amino acid identity, to the enzyme in mycobacteria. The FAS protein is comprised of 3128 amino acids with 7 domains (Figure 1b), six of which catalyze distinct biochemical reactions (Figure 1c). Multiple sequence alignment of Actinomycetales FAS type 1a proteins reveals conservation across all domains (Dataset S1 and Dataset S2). The order of type 1a synthase protein domains, high sequence similarity, and presence of all three key lipid biosynthesis systems (FAS type 1a, MAS-family type 1b, and FAS II) suggest that the substrates and products are similar for the shared enzymatic network of lipid synthases within the suborder Nocardiacae and *Mycobacterium*. A working model for carbon flow in *Rhodococcus* lipid biosynthesis is presented in Figure 1b.

### Enzymes from Expanded Gene Families Generate Lipid Biosynthesis Primers

**Figure 3:**
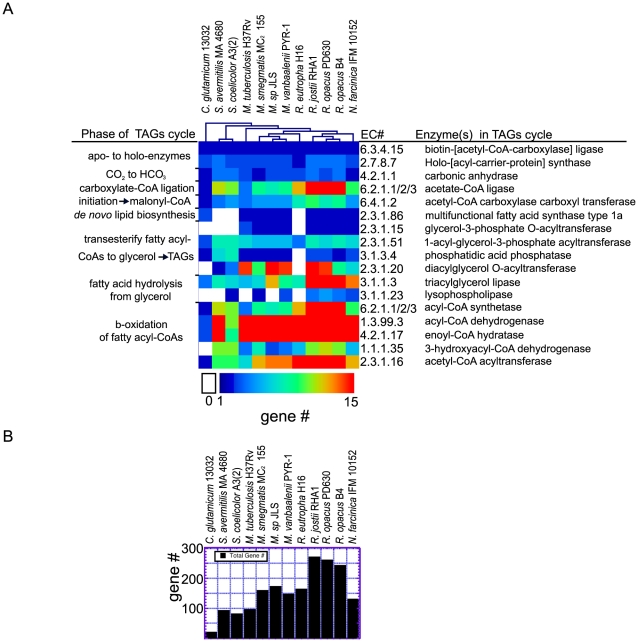
Analysis of genes and gene families implicated in the TAGs cycle. a) The number of genes in the TribeMCL gene-clusters implicated by metabolic reconstruction for each reaction of the TAGs cycle are displayed in a heat map with species clustered according to their TAGs cycle genetic profile. The number of genes corresponding to TribeMCL gene-clusters implicated for each biochemical reaction (EC number) is color coded from 0 to >15. b) The sum of unique genes for each bacterial species implicated for the TAGs cycle by metabolic reconstruction and TribeMCL analysis.

Acetyl-CoA is the product of many catabolic reactions and a key substrate in lipid biosynthesis (http://tinyurl.com/4fgl6zo). Acetate and longer chain carboxylic acids captured from the environment can be converted to -CoA derivatives by CoA synthetases (EC 6.2.1.1) thus feeding these organic acids into lipid metabolism (Figure 1b). The first committed step in lipid biosynthesis is catalyzed by acetyl-CoA carboxylases that function as α/β complexes in *Rhodococcus* and related bacteria of the suborder Corynebacterineae to generate malonyl-CoA that is utilized by FAS type 1a, MAS-family type 1b, and FAS II enzymes for fatty acid biosynthesis (Figure 1b and 1d). Malonyl-CoA is generated by ATP-hydrolysis dependent carboxylation of acetyl-CoA in reaction EC 6.4.1.2 by an expanded family of AccA (α) and AccD (β) enzymes in *Rhodococcus* (Figure 3a). Homologous *Rhodococcus* AccA and AccD proteins were analyzed phylogenetically with those from related species (Figure S4). Some paralogous enzymes from these gene families were reported to recognize the –CoA derivatives of distinct carbon chain length organic acids [31], [32], [33], [34] suggesting that a diverse pool of organic acids could be carboxylated and incorporated into cellular lipids by *Rhodococcus*.

The pool of propionoyl-CoA in *Rhodococcus* can be converted into methylmalonyl-CoA via ATP dependent carboxylation by propionoyl-CoA carboxylase in reaction EC 6.4.1.3 (Figure 1d). In *Mycobacterium* and *Streptomyces* methylmalonyl-CoA is a lipid biosynthetic substrate that is incorporated into methyl-branched lipids by the type 1b fatty acid synthases, a member of the mycocerosic acid synthase MAS-family of proteins [35]. OPAG_06239 is homologous (41% identity over 3527 amino acids) to a protein within the MAS-family with all of the same twelve functional domains and common domain architecture as PKS12 (Figure 1d), suggesting a related role in branched-lipid biosynthesis that was shown in *M. tuberculosis* to be production of C_30–34_ length methyl-branched phospholipids containing a 4,8,12,16, 20-pentamethylpentacosyl lipid subunit [36]. Only one MAS-family type 1b gene is encoded in *R. opacus* PD630 and related *R. opacus* B4, and *R. jostii* RHA1 genomes; whereas in the genera *Mycobacterium*, *Frankia*, and *Streptomyces* expansion of this gene-family has occurred (Figure 2b). In *Mycobacterium* the MAS-family synthases generate methyl-branched lipids adding to the diversity observed in their cell wall lipids that have been shown to be key in persistence, pathogenicity, and immune recognition [37], [38], [39]. By contrast, *Streptomyces* also contain several MAS-family proteins but do not encode for the FAS type 1a protein (Figure 1a). *Streptomyces* are rich in methyl-branched lipids [40], [41] that are shorter than the methyl-branched lipids observed in *Mycobacterium* indicating a difference in the biosynthetic workload between MAS-family and FAS II genes that could result from the absence of the type 1a synthase gene in *Streptomyces*.

### Large Expansions of Gene Families Implicated in the *Rhodococcus* TAGs Cycle of Biosynthesis and Catabolism

The TAGs cycle includes twenty distinct enzymatic reactions starting from acetyl-CoA. The biochemical details of this cycle are presented with the corresponding EC number (Figure S5)(http://tinyurl.com/TAGs-cycle). The large expansions in homologous genes implicated in the TAGs cycle we identified in our initial metabolic reconstruction led us to further analyze the gene-families. We grouped the implicated TAGs cycle genes and families of genes based on protein similarity using the TribeMCL algorithm [42] on a small set of related bacterial species. We found that the genus *Rhodococcus* was deeply enriched in TAGs cycle genes including gene-families of very different sizes and there were no metabolic deficiencies in the multi-step metabolic-cycle (Figure 3a). 261 candidate *R. opacus* PD630 TAGs cycle genes were identified for the TAGs cycle reactions (Figure 3b). The largest gene family in the *Rhodococcus* TAGs cycle corresponds to the FAD dependant acyl-CoA dehydrogenases that operate in the β-oxidation of fatty acyl-CoAs (EC 1.3.99.3). *R. opacus* PD630 contains 71 of these acyl-CoA dehydrogenase genes whereas *Corynebacterium glutamicum* 13032 only contains two genes predicted for this reaction (Figure 3a). Large gene families identified in the *Rhodococcus* TAGs cycle also include: DGATs (EC 2.3.1.20) resolved in a phylogenetic tree (Figure S6), TAG lipases (EC 3.1.1.3), acyl-CoA synthetases (EC 6.2.1.3), enoyl-CoA hydratases (EC 4.2.1.17), and acetyl-CoA C-acyltransferases (EC 2.3.1.16).

*Rhodococcus* species contained at least 261 genes that could contribute to this metabolic cycle without consideration of membrane transport genes as they have yet to be defined for each catabolic substrate. The TAGs cycle -CoA ligases/synthetases that ligate –CoA with carboxylic acids play a role in initiation as well a β-oxidation of lipids have been grouped into 1 category represented as (EC 6.2.1.1/2/3) because we could not resolve the homologous enzymes that vary in their substrate chain length specificity (acetyl- EC 6.2.1.1, propionoyl- EC 6.2.1.2, acyl- EC 6.2.1.3). The largest number of genes in *R. opacus* PD630 dedicated to the TAGs cycle were mostly attributed to the acetate/acyl-CoA synthetases (EC 6.2.1.1/2/3)(18 genes), acyl-CoA dehydrogenases (EC 1.3.99.3)(71 genes), and enoyl-CoA hydratases (EC 4.2.1.17) (64 genes). These large gene families are central to the β-oxidation pathway suggesting that *Rhodococcus* can catabolize and extract energy through aerobic respiration from a diverse range of carboxylic acids as well as biosynthesize from these compounds a diverse array of lipid products.

The related Actinomycetale *C. glutamicum* 13032 provided stark contrast to the large number of lipid metabolism genes we observed in *Rhodococcus*. *C. glutamicum* 13032 had four pathway holes (Figure 3a) with a total of 19 genes implicated in the TAGs cycle (Figure 3b). A substrate-permissive glycerol-3 phosphate acyl transferase could bypass the EC 2.3.1.15 pathway hole allowing for production of essential phospholipids; however the 3-hydroxyacyl-CoA dehydrogenase in the β-oxidation of fatty acids (EC 1.1.1.35) is missing in *C. glutamicum* 13032, a result that is consistent with the observed deficiency in catabolism of fatty acids described below. *C. glutamicum* 13032 is also missing the DGAT enzyme responsible for the final step in TAGs and WE biosynthesis. A complete table of genes for the twelve species presented in Figure 3 can be found in Table S4.

### Odd-Carbon Straight-Chain Lipid Accumulation in *Rhodococcus*

**Figure 4:**
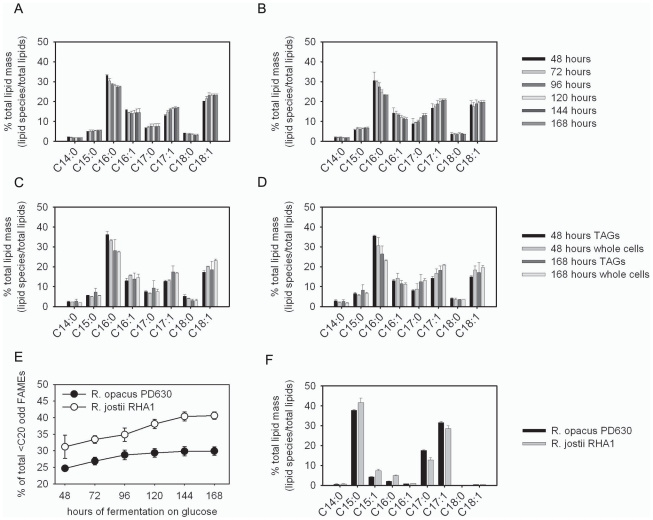
Identification and purification of odd-carbon straight-chain fatty acids generated by *Rhodococcus*. a and b) GC-FID analysis of FAMEs synthesized during fermentation. Freeze-dried whole cells fermented on glucose of *R. opacus* PD630 (a) and *R. jostii* RHA1 (b). c and d) GC-FID analysis of FAMEs derived from TLC-purified TAGs of *R. opacus* PD630 (c) and *R. jostii* RHA1 (d) grown on glucose e) odd lipids C_15∶0_, C_17∶0_, and C_17∶1_ increase during fermentation of both *R. opacus* PD630 and *R. jostii* RHA1. f) *Rhodococcus* grown on propionate generates mostly C_15∶0_, C_17∶0_, and C_17∶1_ fatty acids that were used for purification and confirmation by mass spectrometry and structural ^1^H-NMR. These analyses demonstrated that the purified *Rhodococcus* odd-carbon fatty acids were straight-chain.

During fermentation of glucose, *R. opacus* PD630 and *R. jostii* RHA1 produced abundant lipids that were likely odd in carbon number based on their elution profile on gas chromatograph-flame ionizing detector analysis (GC-FID) (Figure 4a and 4b). The odd-carbon lipid species were found in *Rhodococcus* TAGs that were purified by thin layer chromatography (TLC) prior to conversion into fatty acid methyl esters (FAMEs) for assay by GC-FID (Figure 4c and 4d). During growth on glucose these odd-carbon fatty acids increase in relative abundance accumulating to as much as 30% in *R. opacus* PD630 and 40% in *R. jostii* RHA1 of the total lipids detected in the GC-FID assay (Figure 4e). Fermentation analytics of media concentrations of ammonium and glucose as well as cellular total fatty acids, and residual dry weight (Figure S7) indicated glucose depletion at 96 hours further stimulated *R. jostii* RHA1 to produce higher levels of odd-carbon lipids than *R. opacus* PD630.

### Chemical Identity and Structure of Straight-Chain Odd-Carbon Lipids Stored in TAGs

The FAS type 1a enzyme purified from *Mycobacterium phlei* was shown to convert malonyl-CoA and the C_3_ substrate propionoyl-CoA into undefined fatty acids without propionoyl-CoA carboxylase *in vitro* [27], suggesting that straight-chain odd-carbon lipids could be made by the type 1a FAS. We analyzed the chemical identity and structure of the *Rhodococcus* stored odd-carbon fatty acids to evaluate whether these lipids are methyl-branched or straight-alkyl chains in order to provide insight into the enzyme(s) responsible for this uncommon lipid biosynthesis. The known substrate preferences of the FAS type 1a enzymes are straight-chain substrates (acetyl-CoA, propionyl-CoA, and malonyl-CoA [26], [27]) while the MAS-family type 1b synthases incorporate the C_3_ methyl-branched lipid substrate methylmalonyl-CoA [35], [37], [39], [43], [44].

To determine the identity and chemical structure of the putative *Rhodococcus* odd-carbon lipids we purified them as FAMEs by taking advantage of their enrichment when *R. opacus* PD630 and *R. jostii* RHA1 were grown on propionate as the sole carbon source (Figure 4f). Propionate can be converted intracellularly to propionoyl-CoA through the activity of propionoyl-CoA ligase (EC 6.2.1.2)(Figure 1b) allowing for degradation via the methylcitrate cycle [45] or incorporation into lipids via two routes. In the straight-chain lipid biosynthesis pathway diagramed in Figure 1b, propionoyl-CoA is a substrate in the initial condensation reaction with malonyl-ACP. By contrast, in the branched-lipid biosynthesis pathway diagrammed in Figure 1d, propionoyl-CoA is first converted into methylmalonyl-CoA prior to incorporation into lipids by the MAS-family type 1b synthases.

FAMEs from propionate grown cells were purified via reverse phase HPLC then analyzed on a coupled gas chromatograph/electron ionization-mass spectrophotometer GC-EI-MS. We identifed ions with masses that corresponded to the methyl esters of pentadecanoic acid m/z 256 Da C_15∶0_ (Figure S8), heptadecanoic acid m/z 284 Da C_17∶0_ (Figure S9), and heptadecenoic acid m/z 282 Da C_17∶1_ (Figure S10). Fragmentation of these full length FAMEs resulted in ions that also matched previously reported spectra for electron ionized ions with these odd-carbon chain length FAMEs [46].

To discriminate between structural isomers with the same molecular weight (straight-chain and methyl-branched lipids), we performed ^1^H-NMR on the HPLC-purified FAMEs from *Rhodococcus* (Figure S11). We saw evidence of the methyl esters as expected from the transesterification of cellular lipids with methanol at 3.68 ppm; however, the FAMEs purified from *Rhodococcus* showed no evidence of methyl-branching at 0.86 ppm along the aliphatic chain of the C_15∶0_, C_17∶0_, and C_17∶1_ fatty acids as compared to the branched control 16-methylheptadecanoate. The NMR spectra in addition to the mass spectra demonstrate that these odd-carbon lipids contained straight-chain alkanes and a cis-alkene, not the methyl-branched forms expected from a methylmalonyl-CoA intermediate generated by propionoyl carboxylase in reaction EC 6.4.1.3. The absence of methyl-branched lipids in the shorter odd-carbon storage lipids indicated that *de novo* biosynthesis of methyl-branched lipids in *Rhodococcus* if present is restricted to the longer chain length lipids as previously described for the cell wall associated lipids in *Mycobacterium*. The identification of the stored pentadecanoic acid, heptadecanoic acid, and heptadecenoic acid odd-carbon straight-chain fatty acids could result from the FAS type 1a enzyme that is known to incorporate the three carbon molecule propionoyl-CoA and malonyl-CoA [27]. The odd-carbon fatty acids isolated from *Rhodococcus* grown on glucose contained predominantly seventeen carbons, that is in the range where most FAS type 1a products are released from the type 1a FAS (C_16–18_) [26].

### Catabolic Phenotyping of Four Industrially Utilized Bacterial Species

*Rhodococcus* fermentation of low-cost organic substrates into oil requires a more complete understanding of the catabolic capabilities of these species. We tested 190 organic compounds as the sole carbon source in time course growth assays with four soil-derived bacterial species including the Actinomycetales *R. opacus* PD630, *R. jostii* RHA1, *Corynebacterium glutamicum* 13032, and the Gram-negative β-proteobacterium *Ralstonia eutropha* H16. Four chemical categories of compounds including carboxylic acids, nitrogen containing, carbohydrates and alcohols, and oligosaccharides were tested in these bacterial time course growth assays. Compounds capable of supporting growth yielded growth values that were clustered hierarchically to show catabolic-relationships between growth substrates and the species being compared.

**Figure 5:**
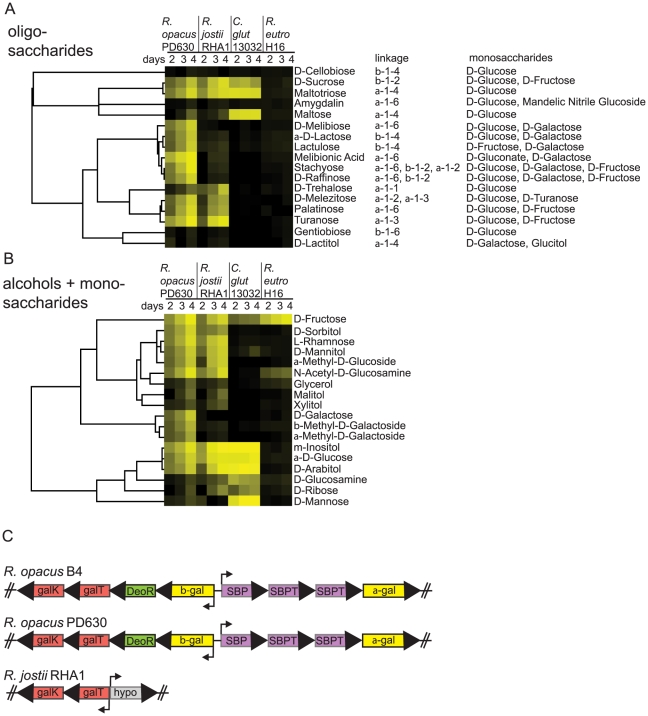
Screens of four bacterial species for growth on carbohydrates and alcohols. a) Compounds were clustered according to how *R. opacus* PD630, *R. jostii* RHA1, *C. glutamicum* 13032, and *R. eutropha* H16 were able to grow on oligosaccharides from 2–4 days. Yellow indicates evidence of growth. b) alcohol and monosaccharide compounds were clustered according to bacterial growth as in a. c) Comparison of three *Rhodococcus* chromosomes revealed that *R. opacus* B4 and *R. opacus* PD630 shared two divergent operons dedicated to galactose and oligogalactoside metabolism but *R. jostii* RHA1 only had a small piece of this chromosomal region containing the GalK and GalT genes omitting α- and β-galactosidases, Solute Binding Protein (SBP), two Solute Binding Protein Transporter (SBPT) proteins, and a DeoR family transcriptional regulatory protein.

Carbohydrates and alcohols are an important class of compounds to evaluate for converting natural organic streams such as cellulose and cellulose derived sugars, sugarcane, and beet sugars into biofuels. Seventeen oligosaccharides were screened for growth resulting in the identification of 13, 8, 3, and 0 growth substrates for *R. opacus* PD630, *R. jostii* RHA1, *C. glutamicum* 13032, and *R. eutropha* H16 respectively (Figure 5a). *R. eutropha* H16 showed no ability to degrade the oligosaccharides tested and relatively few monosaccharides were catabolized indicating that this species has many pathways to catabolize organic streams such as the rich pool of carboxylic acids defined below or fix CO_2_ by using the energy derived from splitting H_2_ [47], [48], [49]. *Corynebacterium* and *Rhodococcus* catabolized the disaccharides sucrose and maltose as well as the trisaccharide maltotriose. *Rhodococcus* species grew poorly on disaccaharide maltose but well on the trisaccharide maltotriose suggesting a possible membrane-transport preference. *R. opacus* PD630 gene OPAG_05551 is a glycogen hydrolase that contains the predicted activity to account for maltotriose growth that are part of an operon containing OPAG_05551 that breaks down glycogen (glucose α1–4) with P_i_ to yield glucose-1-P. Glucose 1-P isomerase is also utilized in galactose catabolism to generate the glucose 6-P that is common to glycolysis, pentose phosphate, and Entner Duoderoff catabolic pathways that are all complete pathways in our sequence based metabolic reconstructions of *Rhodococcus* (http://tinyurl.com/GLC-6-P).

### Galactose Metabolism Distinguishes *Rhodococcus opacus* PD630

The compound-specific growth assays indicated that galactose and oligogalactoside metabolisms differ between *R. jostii* RHA1 and *R. opacus* PD630 (Figure 5b). The ability of *R. opacus* PD630 to metabolize galactose enables efficient growth on the oligogalactosides lactose, lactitol, melibionic acid, melibiose, lactulose, raffinose, and stachyose (Figure 5a). A search of our metabolic reconstruction for the genetic basis of the galactose phenotypic differences between *R. opacus* PD630 and *R. jostii* RHA1 led to identification of a galactose-catabolic region of *Rhodococcus* genomes that is shared between the two *R. opacus* species B4 and PD630, but not in the closely related *R. jostii* RHA1 species. Two divergent polycistrons in the galactose catabolic region are fully syntenic between the *R. opacus* PD630 and *R. opacus* B4 species that contain the hydrolytic α- and β-galactosidases as well as solute binding protein (SBP), solute binding protein transporters (SBPT), and transcription regulatory protein of the DeoR family (Figure 5c) that could collectively hydrolyze and transport mono- and oligo-galactosides supporting growth on these compounds.

### *Rhodococcus* Degrades a Diverse Pool of Carboxylic Acids

**Figure 6:**
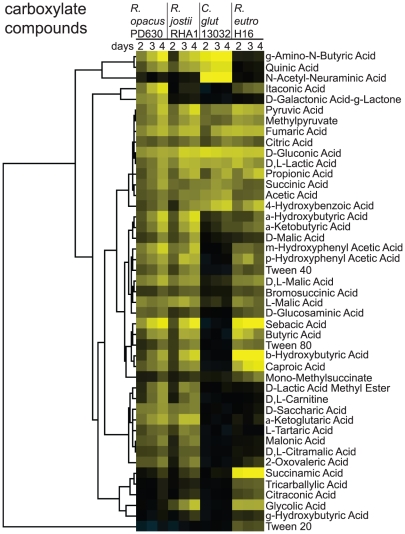
Screens of four bacterial species for growth on carboxylate compounds (organic acids). Compounds were clustered as in Figure 5a.

Carboxylic acids appear to be an important carbon source for *Rhodococcus* and *R. eutropha* H16. To determine the utilization of carboxylic acids as carbons source, we tested 64 carboxylic acids for growth using the previously described assay (Figure 6). These analyses led to the identification of 39 growth substrates for *R. opacus* PD630 and *R. jostii* RHA1 (61% of carboxylates tested), 15 growth substrates for *C. glutamicum* 13032 (21% of carboxylates tested), and 34 growth substrates for *R. eutropha* H16 (53% of carboxylates tested)(Figure 6). Acetic and propionic acids support growth in all species tested as did other common intermediates in central metabolism such as pyruvic, succinic, and citric acids. All of the species tested grew on gluconic acid that is degraded by the pentose phosphate pathway.

Similar to the observed galacto-saccharide phenotypes, *R. opacus* PD630 uniquely degrades D-galactonic acid-g-lactone (Figure 6). By contrast, growth on the short chain hydroxy acid glycolic acid is specific to *R. jostii* RHA1 and *R. eutropha* H16 (Figure 6). Growth was observed for the longer chain α and β hydroxybutyric acids by both *Rhodococcus* species and *R. eutropha* H16, whereas weak growth on γ hydroxybutyric acid was again only seen for *R. jostii* RHA1 (Figure 6) suggesting that this species has a more diverse hydroxy-acid metabolism.

*R. opacus* PD630, *R. jostii* RHA1, and *R. eutropha* H16 grew well on the C_10_ dicarboxylate sebacic acid suggesting that β-oxidation of these longer chain dicarboxylates provides rapid growth. β-oxidation could also explain the growth on Tween 40 and Tween 80 compounds by *Rhodococcus* and *Ralstonia* (Figure 6). The Tween compounds are converted to fatty acids upon ester-hydrolysis by cutinase proteins encoded for in the genomes of Actinomycetales [50]. Tween 20 supported growth of only *R. eutropha* H16 likely due to toxicity for the other species resulting from the hydrolytic release of a C_12_ fatty acid. *C. glutamicum* 13032 is limited in carboxylates catabolism as seen by the inability to catabolize sebacic acid and the longer chain Tween 40 and Tween 80 (Figure 6). A limited carboxylate catabolic-profile observed for *C. glutamicum* 13032 in our growth assay is consistent with genes missing for 3-hydroxyacyl-CoA dehydrogenase within the fatty acids β-oxidation pathway (Figure 3a).

### *C. glutamicum* Degrades Sialic Acid

The identification of the sialic acid or *N*-acetylneuraminic acid, an abundant component of extracellular glycoproteins, as a growth substrate for the soil bacterium *C. glutamicum* 13032 (Figure 6) was unexpected because this pathway has only been described for bacteria that colonise animals [51], [52]. Examination of the *C. glutamicum* 13032 genome reveals a likely sialic acid catabolic operon containing genes for a secreted sialidase (*cg2935*), a sialic acid ABC transporter (*cg2937–2940*) of the *satABCD* type seen in *Haemophilus ducreyi* [*53*] and a full set of catabolic genes (*cg2928–9* and *cg2931–3*) genes that would allow *C. glutamicum* to degrade sialic acid to fructose-6-phosphate, pyruvate, and ammonia [51]. A related but incomplete catabolic operon is seen in *C. diptheriae* NCTC 13129. The sialic acid catabolic genes are not conserved in the other species tested nor were related transporters shown to facilitate uptake of sialic acid [54], consistent with these phenotypic results.

### Diverse Nitrogen Metabolism in *Rhodococcus*

*Rhodococcus* displayed the most diverse catabolism of nitrogenous compounds in our species-comparative time course growth assays. Clustered heats maps relating the time course growth of 38 nitrogenous compounds tested. 26 nitrogenous compounds supported growth in at least one of the species tested (Figure S12). A notable feature of the *Rhodococcus* species nitrogenous metabolism is the ability to catabolize the branched amino acids that result in the formation of propionoyl-CoA thereby establishing a cycle of propionoyl-CoA in carbon storage and amino acid catabolism (http://tinyurl.com/propionyl-CoA). A ranked order list of growth values on 190 compounds for each species in the screen is presented in Table S2.

### Expanded Families of Membrane Transport Proteins in *R. opacus* PD630

Comparison of the protein domains (Pfam database, Sanger Institute UK) within a set of related Actinomycetales and the Gram-negative outlier *R. eutropha* H16 revealed that the *R. opacus* PD630 genome contained 3 membrane transport protein families in the top 9 expanded families of total encoded protein domains (Figure S13). The membrane transport major facilitator super family (MFS) is the most prevalent Pfam domain in *R. opacus* PD630. There are 229 MFS genes in *R. opacus* PD630 as compared to 176 in *R. jostii* RHA1, 104 in *R. eutropha* H16, and 47 in *C. glutamicum* 13032 (Figure S14). The high relative number of membrane transporters likely enables the broad catabolism we observed in *R. opacus* PD630 and *R. jostii* RHA1.

### Cholesterol Degradation by a Large Genomic Region

The ability to degrade sterols is shared between *Rhodococcus* and *Mycobacterium*. Following intravenous injection, *M. tuberculosis* has been observed to colonize lung tissue that is rich in lipid bodies and cholesterol crystals [55]. Genetic analysis of *M. tuberculosis* cholesterol catabolic pathways showed this sterol to be an essential carbon source during *M. tuberculosis* infections in mouse lung models [56], [57], [58]. Complete sterols degradation requires catabolism of both of the aliphatic branched side chain as well as the terpene polycyclic rings; however catabolism studies of *M. tuberculosis* showed that ^14^C labeling at the fourth position of the steroid A ring was released as CO_2_; whereas the label at position 26 within the sterol branched side chain was converted into phthiocerol dimycocerosate (PDIM) [57]. This study indicated that part of the sterol was being degraded for energy and the other was being used for assembly of cell wall associated branched-lipids. Sterol A and B ring degradation results in propionoyl-CoA and pyruvate while side chain degradation results in a 2∶1 formation of propionoyl-CoA: acetyl-CoA. In *M. tuberculosis* the the propionoyl-CoA from cholesterol degradation is converted into methylmalonyl-CoA then incorporated into branched-fatty acids such as PDIM by the PKS12 MAS-family type 1b synthase [35], [37], [43], [44], [59].

A large region (∼0.28 Mb) of *Rhodococcus* chromosomal DNA has been identified through transcriptomic and genetic analysis of *R. jostii* RHA1 grown on cholesterol as a sole source of carbon [60]. Within this large chromosomal region, there are six clusters of genes that encode for the multiple enzymes dedicated to sterol degradation including: membrane transport, side chain degradation, sterol A and B ring degradation, and sterol C and D ring degradation, for recent review [61]. We performed whole genome alignments between *R. opacus* PD630, *R. jostii* RHA1, and *R. opacus* B4, *M. sp* JLS, *M. vanbaalenii* PYR-1, *M. smegmatis* MC^2^ 155, *M. tuberculosis* H37Rv, *C. glutamicum* 13032, and *S. avermitilis* MA 4680 to evaluate gene conservation within the six gene clusters implicated in cholesterol degradation (Figure S14). We found extensive conservation in the cholesterol degradation genes between *Mycobacterium* and *Rhodococcus* as has been reported previously [60]. Our analysis indicates *Streptomyces* contains many of the key genes for cholesterol degradation but is lacking homologues to the Mce sterol-transport genes [57]. *C. glutamicum* 13022 did not contain the genes implicated for cholesterol degradation. The only major difference found between the previously described sterol degradation chromosomal region in *R. jostii* RHA1 and *R. opacus* PD630 is the presence of a transposase gene in gene cluster 2 (OPAG_09155).

## Discussion

The *Rhodococcus* genomes encode multiple biosynthetic pathways for making lipids and expanded gene families within those pathways that contribute to the diversity and abundance of lipid products seen with some Actinomycetales. We demonstrated that *Rhodococcus* TAGs contain the uncommon straight-chain odd-carbon lipids pentadecanoate, heptadecanoate, and heptadecenoate by mass and structural determination. The high abundance of the straight-chain odd-carbon lipids are yield-controllable through feeding of the three carbon organic salt propionate. Propionate is converted to propionoyl-CoA, a metabolite that is generated during the catabolism of the branched amino acids isoleucine, valine, and threonine as well as sterols. Propionoyl-CoA levels likely increase intracellularly during nitrogen and glucose starvation resulting from elevated protein and amino acid degradation as the physiology of these cells adapt to these nutrient limitations. Elevated propionoyl-CoA has been proposed to explain increased production of the branched-lipid PDIM when mycobacteria is grown on cholesterol as a sole source of carbon [59]. The FAS type 1a protein in the genus *Rhodococcus* is highly related to the mycobacterial protein that has been demonstrated to incorporate propionoyl-CoA during *de novo* lipid biosynthesis and produce lipids with the same lengths observed in the stored lipids TAGs and WEs. Our genomic analysis indicates conservation of all three interconnected lipid biosynthesis systems in *Nocardiacae* and *Mycobacterium* namely FAS type 1a, MAS-family type 1b, and FAS II. This group of bacteria also shares the ability to store lipids in high abundance. This conserved lipid biosynthetic network provides means for extrapolating functional information derived from decades of lipid metabolism research in *Mycobacterium* to the elaborate lipid metabolism in *Rhodococcus*.

Pharmacological studies in combination with enzyme activities performed with purified systems demonstrated that *Mycobacterium* uses FAS type 1a to convert two and three carbon organic acids into straight-chain fatty acids containing 16, 18, 24, and 26 carbons [20], [26], [27]. FAS type 1a products are released as the –CoA derivative of a fatty acid due to the dual activity of the FAS type 1a malonyl palmitoyl transferase (MPT) domain [21]. The FAS acyl-CoA products can then proceed through multiple lipid biosynthetic pathways. Studies of mycobacterial lipid metabolism have established a genetic model for this related oleaginous bacterium that connects multiple biosynthetic systems for the building of storage, plasma membrane, as well as very long chain cell wall-associated lipids. The close phylogenetic relationship between the genus *Mycobacterium* and *Rhodococcus* resulted from common ancestry wherein these genera share many features of their elaborate lipid metabolisms.

Much of what is known about Actinomycetales odd-carbon lipid metabolism comes from work that described longer chain extracellular lipids that are methyl-branched. Prior to this study, it was unclear whether the <C_20_ odd-carbon lipids in *Rhodococcus* are methyl-branched as a result of incorporating methylmalonyl-CoA [16]. Using ^1^H-NMR to analyze purified lipids we could distinguish between the structural isomers of branched and straight-chain fatty acids demonstrating that the stored odd-carbon lipids in *Rhodococcus* are straight-chain. The odd-carbon storage lipids in *Rhodococcus* could result from propionoyl-CoA and malonyl-ACP condensing in the initiation phase of FAS type 1a biosynthesis (Figure 1b). The substrate requirements for production of straight-chain odd-carbon lipids matches the reported substrate specificity of the multifunctional FAS type 1a enzyme and its product chain lengths. The combination of metabolic product identification and metabolic pathway mapping through genomic sequence supports a serial order to the elaborate network of lipid biosynthesis in *Rhodococcus*, similar to *Mycobacterium*, wherein FAS type 1a initiates and elongates fatty acids releasing –CoA products that are in the range of C_16_–C_18_ and C_24_–C_26_. FAS produced fatty acyl-CoAs can be further elongated in the type II system or by the MAS-family type 1b synthase.

The *Rhodococcus* type 1b synthase OPAG_06239, like the homologous PKS12 gene from *Mycobacterium*, contains two modules consisting of 6 protein domains in a direct repeat (Figure 1d). The PKS12 enzyme displays dual specificity for malonyl-CoA and methylmalonyl-CoA within the same polypeptide and oligomerizes in a tail-to-head fashion to perform multiple elongation cycles on C_16_- and C_18_-CoA molecules resulting in C_30–34_ multiply methyl-branched fatty acids. Enzyme assays with single modules (6 domains) of PKS12 and site-specific mutations within the PKS12 acetyl transferase (AT) domains demonstrated that the N-terminal module incorporates methylmalonyl-CoA whereas the C-terminal module incorporates malonyl-CoA [43]. This mode of biosynthesis results in branched fatty acids that contain a methyl-branch at every fourth carbon from the point of initial condensation by the type 1b enzyme. This alternating of elgonation substrates mechanism explains the curious methylation at every fourth carbon in the mannosyl-β-1-phosphomycoketide (MPM) molecules isolated from *Mycobacterium* [36]. In *Mycobacterium*, the enzymes of the type 1b MAS-family and FAS II system share a common preference for longer chain length fatty acyl-CoAs resulting in the observed order in biosynthesis enzyme activity. The order of conserved domains and sequence homology of the *Rhodococcus* type 1b synthase OPAG_06239 suggests that this enzyme will function similarly in some aspects of branched lipid biosynthesis that is characteristic of the MAS-family enzymes in the related *Mycobacterium* species; however no methyl-branched lipids have been identified in *Rhodococcus* to date.

In species such as *Mycobacterium* that have both FAS type 1a and 1b synthases, there is a distribution in labor amongst the synthases that is dictated by chain length; wherein FAS type 1a functions as the initiating synthase and the type 1b synthase incorporates methyl branch substrate methylmalonyl-CoA during lipid biosynthesis of longer chain length lipids. In more distantly related Actinomycetales, the genera *Saccharopolyspora*, *Salinspora*, *Frankia*, and *Streptomyces* we observed a type 1b but no type 1a synthase (Figure 1a); thus preventing the distribution of labor observed in Nocardiacae and *Mycobacterium*. *Streptomyces* have been reported to store abundant methyl-branched lipids [41] that are shorter in chain length than those isolated from *Mycobacterium*. The observation of odd-carbon lipids in the TAGs from *Rhodococcus* intrigued us as a possibility that type 1b synthase activity was contributing to the accumulated TAGs. The chemical analysis we performed indicated the stored TAGs were straight-chain lipids of the appropriate length to have been built by type 1a FAS through iterative biosynthesis with two of this enzymes known substrates (propionyl-CoA and malonyl-CoA). How is the strict distribution of labor observed between type 1a and the other type II and 1b synthases maintained in Nocadiacae and *Mycobacterium*? The crystal structure of the related FAS from *S. cerevisae* [62] revealed this synthase is a hexamer complex of apoenzymes. A hexamer complex also explains the behavior in ultracentrifugation studies of the FAS type 1a complex from mycobacteria [26]. The structural studies of fungal FAS provided a structure-based model wherein fatty acids are biosynthesized within the cavity of a 2.6 MDa β barrel structure. The enzyme FAS-ACP domain with growing acyl chain accesses individual catalytic domains shuttling biochemical intermediates from one active site to the next within the FAS type 1a hexamer complex [62]. Fatty acid products of the appropriate length are released as acyl-CoAs by the MPT domain of FAS type 1a. The FAS hexamer in *Mycobacterium* display a bimodal distribution of product chain lengths that are (C_16–18_ and C_24–26_) [26]. We propose the FAS 1a acyl-CoA products become accessible to the other synthases once released from within the β barrel structure of the FAS hexamer by the palmitoyl transferase activity of the MPT domain.

Acetyl/acyl transferase (AT) domains in type 1 fatty acid synthases display remarkable substrate diversity. In the type 1a synthase, the AT domain loads acetyl–CoA on the FAS-ACP domain then is subsequently transferred to a cysteine in the keto synthase (KS) domain. The second substrate for condensation is the elongation substrate malonyl-CoA that is transfered by the FAS-MPT domain to the FAS-ACP domain then delivered to the KS domain for decarboxylating condensation with acetyl-CoA resulting in acetoacetyl-ACP. The type 1b synthases that inititiate fatty acid biosynthesis, like the mammalian FAS, lack the MPT domain thus use their AT domain to load both acetyl-CoA and malonyl-CoA. MAS-family type 1b enzymes display significant substrate diversity by varying both the acyl substrates (acetyl-CoA, acyl-CoAs) as well as elongation substrates (malonyl-CoA, methylmalonyl-CoA). We found seven Actinomycetales genera that contained the MAS-family type 1b synthases compared to 4 genera that encode the type 1a synthase. Six out of seven Actinomycetales genera that encode type 1b synthases also encode at least one storage enzyme of the DGAT/WE family. We conclude the relatively widespread taxonomic representation of the type 1b synthases and the diversity of substrates these enzymes react on suggests that there are lipid variants yet to be identified.

The large number of genes dedicated to lipid metabolism in Actinomycetales is the result of gene duplications, multifunctional FAS type 1a and MAS-family type 1b gene emergence, horizontal gene transfer, and emergence of the DGAT/WE enzymes that catalyze the transesterification of fatty acyl-CoAs with diacylglycerol. We performed phylogenetic analysis on genes identified by metabolic reconstruction in an attempt to predict the most likely activities for each enzyme encoded in the *R. opacus* PD630 genome. We used the TribeMCL algorithm to describe the size of the gene family and all of the family members for a set of related Actinomycetales. Phylogenetic tree formation with these related species was used to resolve the orthologous from paralogous enzymes, thus facilitating the extrapolation of functional data for related proteins to our *in silico* model of *Rhodococcus* Opacuscyc14.5. The broad catabolism and elaborate lipid biosynthesis described in *Rhodococcus* indicates that there is substantial enzymatic activity on chemically-related compounds that could be explained by expansion in gene-family sizes and genetic drift to encode enzymes with slightly variant substrate recognitions from ancestrally-related enzymes but still catalyze similar chemical reactions. The 8632 genes in *R. opacus* PD630 provide a large arsenal of metabolic enzymes for an oleaginous lifestyle within soil. We found protein domains contained in transport proteins to be the most abundant class of domains encoded in *Rhodococcus* as well as a distinctive profile of transporter types that are distinguishing for *Rhodococcus* (Figure S13). Identification of structural genes and operons that play a role in the lipid body assembly process have already begun to benefit from genomic sequence [63].

Chemoheterotrophic organisms capable of fermenting sugars and a broad spectrum of organic compounds derived from cellulosic and other natural resource biomasses through bioconversion provides an industrial process to convert agricultural side-, natural resource-, and industrial waste-streams into fungible fuels. *R. opacus* PD630 differs from *R. jostii* RHA1 in its ability to catabolize the cellulosic sugar galactose and oligogalactosides. Both *Rhodococcus* species degraded the cellulosic sugars glucose and rhamnose; however, none of the species tested were able to degrade the disaccharide cellobiose containing β1–4 linked glucose. Cellulose catabolism requires hydrolysis of β1–4 linked glucose, indicating that these species do not encode for these hydrolases. The most abundant component of hemicellulose is xylan that is broken down to xylose. Consistent with the cellobiose deficiency these species were unable to catabolize xylose. Genetic complementation of cellulosic degradation pathways in *Rhodococcus* provides a streamlined approach for cellulosic biomass conversion into oil-based fuels. *Rhodococcus* species catabolized a diverse array of carboxylic acids that corresponded to an expansion in the acyl-CoA ligase gene family that link organic acids with biosynthesis and catabolism. Organic acids are produced in the mixed-acid fermentations of cellulose degrading organisms thus indicating a potential next-generation strategy for 2-phase fermentations of cellulosic biomass to TAGs that could be done without genetic modification.

Our working model of the *R. opacus* PD630 metabolism began with genome sequence that allowed phylogenetic comparisons to be made with related species that have been studied in far greater molecular detail. Phenotypic information about catabolism in *Rhodococcus* provided a powerful multigenic test that guides the metabolic reconstruction towards completion through phenotype-directed pathway curation. Literature-based pathway curation united the reported biochemical reactions of *M. phylei* FAS type 1a protein with metabolic products that we purified and characterized during metabolic model refinement of *R. opacus* PD630. The improvements to our genetic model of *R. opacus* PD630 metabolism provides a template for further refinement with the integration of data from genetics, biochemistry, metabolomics, lipidomics, and transcriptomics that will be the focus of future work.

## Materials and Methods

### Bacterial Species Used in This Study

*R. opacus* PD630 was obtained from the DSMZ strain 44193. *R. eutropha* H16 ATCC17699 and *C. glutamicum* ATCC13032 were from ATCC. *R. jostii* RHA1 was a gift from Lindsay Eltis at the University of British Columbia.

### *Rhodococcus opacus* PD630 DNA Sequencing and Genome Annotation

Genomic DNA was extracted from *R. opacus* PD630 as in [64] without the addition of mutanolysin. Genomic DNA was sequenced at 454 Life Sciences (A Roche Company) according to manufacturer protocol for shotgun (Rapid Library) and 3 kb paired-end reads using GS FLX Titanium sequencing chemistry. The resulting DNA sequence was assembled using the GS *De Novo* Assembler software version 2.0. Open Reading Frames (ORFs) were predicted from assembled genome sequence using a combination of *in silico* ORF predictions and gene mapping from the annotated *R. jostii* RHA1 genome. *In silico* ORFs were predicted using GeneMark [65] and Glimmer3 [66]. For synteny based gene-mapping Nucmer [67] was used to find local alignments between every contiguous sequence read (contig) in the *R. jostii* RHA1 and every contig in the *R. opacus* PD630. A whole-genome synteny map was then built from these local alignments by chaining together collinear hits, allowing up to 10,000 bases of undetectable similarity between anchors, then filtering out chains that overlap a larger chain on either sequence by more than 90% of their length. The remaining chains, corresponding to syntenic regions of DNA, were globally aligned using LAGAN [68]. Each transcript in the *R. jostii* RHA1 genome was mapped onto the *R. opacus* PD630 by attempting two mapping techniques and then selecting the transcript with the higher-scoring global alignment to the reference transcript's coding sequence. The first method projects the boundaries of each gene onto the target genome using the coordinates in the raw whole-genome alignment. The second method uses the whole-genome alignment to define a target region containing the reference transcript, and then uses GeneWise to build a gene model by aligning the reference protein to the target region. Final ORFs were defined by comparison of *in silico* ORFs and mapped ORFs with hits to Pfam [69] and the top blast hits against the non-redundant protein database. ORFs with overlap to non-coding RNA features (see below) were reviewed and removed when appropriate. Discrepancies in the final ORFs were resolved via manual review. Ribosomal RNAs (rRNAs) were identified with RNAmmer [70]. The tRNA features were identified using tRNAScan [71]. Other non-coding features were identified with RFAM [72]. Every annotated gene in the *Rhodococcus opacus* PD630 genome is assigned a locus number of the form OPAG_##### both at the Broad Institute web site and in GenBank with accession ABRH01000000.

### Stitching the Genome Assembly Based on Synteny

The *R. opacus* PD630 V3 genome assembly contained 293 (contigs), containing 16 scaffolds longer than 2,000 residues. The 16 large scaffolds of the *R. opacus* PD630 genome, 10 of which were largely syntenic with *R. jostii* RHA1 and *R. opacus* B4 linear chromosomes, were ordered and oriented to reflect the observed synteny followed by the smaller contigs. Nucmer and mummerplot, components of the mummer 3.0 software package [67], were used to align the genomes and create graphical representations of those alignments. The mummerplot graph files were annotated to show boundaries between each of the scaffolds in the mummerplot graphs. Through manual inspection of these graphs, the syntenic order and orientation of these contigs were assembled into a stitched FASTA file with the addition of 500 N nucleotides at scaffold termini.

### Metabolic Reconstruction

Enzymes for nine comparative species in Opacuscyc14.5_compare were computationally predicted using the EFICAz2 algorithm, enzyme prediction using gene-name matching, pathway prediction [24], transcription unit prediction [73], transporter prediction [74], and pathway hole filling [75] was performed with the Pathway Tools 14.5 software [24]. These databases can be accessed at (http://rhodocyc.broadinstitute.org). In addition, Opacuscyc14.5_working also predicted enzymes using homology to proteins with an EC# assignment in the database at Kyoto Encyclopedia of Gene and Genomes KEGG (http://www.genome.jp/kegg/). Subsequent literature-based manual curation was used to refine Opacuscyc14.5 TAGs biosynthesis and degradation cycle.

### Phylogenetic Analysis of FAS, MAS, PKS12, and DGAT Gene Families

A set of directed gene pairs was generated by performing an all-against-all BLASTP search (min % aligned = 10 and e-value<1e^−5^) between a comparative set of genomes (all accession numbers available in Table S3). Genes were clustered using OrthoMCL [76] with a Markov inflation index of 1.5 and a maximum e-value of 1e^−5^. Gene clusters were identified to which the *M. tuberculosis* FAS, MAS, PKS12, and DGAT genes belonged to. These clusters had all members plotted on the AMPHORA phylogenetic tree of the Actinobacteria [28]. In the phylogenetic analysis of FAS genes, we included three fungal FAS genes and two stramenopile FAS genes, while in the phylogenetic analysis of MAS-family genes we added four animal FAS genes. Amino acid sequences were aligned using MAFFT [77] using the E-INS-i method. A maximum likelihood phylogeny was estimated using the PROTGAMMABLOSUM62 model in RAxML [78] with 1000 bootstrap replicates. FAS type 1a protein alignments were visualized using Jalview [79] and domains were annotated based on predictions from the Conserved Domain Database [80].

### Phylogenetic Resolution of Acc and DGAT Proteins

A custom blast database containing seven *Rhodococcus* & *Mycobacterium* genomes, including *R. opacus* B4, *R. opacus* PD630, *R. jostii* RHA1, *M. tuberculosis* H37Rv, *M. vanbaalenii* PYR-1, *M.* sp JLS & *M. leprae* Br4923 was generated. Protein sequences annotated as either AccA or AccD within *Mycobacterium tuberculosis* H37Rv were identified and accessions extracted into separate lists, whereas the WS/DGAT protein accessions were identified from the *R. jostii* RHA1 genome annotation. Each set of amino acid sequences were blasted against the database using BLASTP with an expect threshold of 1E^−30^. Unique matches were identified and whole sequences extracted for alignment with MUSCLE v3.7 [81], using a maximum of 24 iterations. Alignments were manually checked with ClustalX v2.0 [82], at which point WS/DGAT sequences without any residues aligning to the proposed active site motif (H[L/S/P]xxxDG) [4] were rejected. The multiple sequence alignments were converted to phylip format for passing to ProtTest v2.4 [83], which determines the best-fit substitution model and produces a phylogenetic tree with maximum likelihood estimation, using PhyML v3.0 [84]. Newick formatted trees were represented with iTOL [85].

### Mass and Structural Analysis of Odd-Carbon Lipids

GC-MS analyses were conducted on a TraceGC Ultra DSQ mass spectrometer (Thermo Scientific) equipped with an AT-5 ms column from Alltech (60 m×0.25 mm i.d. ×0.25 mm d_f_). The injector and transfer line were maintained at 280°C while the ion source was set at 180°C in electron ionization (EI) mode. High purity helium was used as carrier gas at a flow rate of 1 mL/minute. The sample was injected onto the column in split mode and heated at 40°C for a minute. The GC oven temperature was increased to 280°C at 20°C per minute.

The NMR spectra were recorded on a Varian Inova instrument, operating at 500 MHz for ^1^H and 125 MHz for ^13^C, equipped with a three channel, 5 mm, indirect detection probe, with z-axis gradients. The solvent was chloroform-*d*, and the temperature was 25°C. The chemical shifts for ^1^H were referenced to the residual solvent signal, 7.27 ppm on the tetramethylsilane scale. Proton spectra were acquired in 4 transients, with a 30° pulse, an acquisition time of 5 s and no relaxation delay. The intensity of the signals was referenced to the signal of the terminal methyl in the alkyl chain, at 0.88 ppm, 3H.

### Purifications of Odd-Carbon Lipids

100 ml cultures of *R. opacus* PD630 and *R. jostii* RHA1 were grown in defined media [5] with substitution of 1% propionate/0.056% NH_4_SO_4_ for 1 week at 30°C with agitation followed by addition of 1 gram of propionate from sterile 20% stock solution. 50 mls of culture was collected after 2 weeks by pelleting and freeze dried prior to lipid extraction. 50 mg of dried cells were transesterified in a 2 ml volume of 50% CHCl_3_/42.5% methanol/7.5% H_2_SO_4_ for 2.5 hours at 100°C in sealed 16×125 ml glass tubes (Kimble Glass Co.). *Rhodococcus* fatty acid methyl esters (FAMEs) preps were then concentrated under a stream of N_2_, resuspended in dichloromethane (DCM), and analyzed (10-mL injections) using an Agilent 1050 High Performance Liquid Chromatography (HPLC; Agilent Technologies, Inc, Wilmington, DE USA) coupled to a Wakosil II RS-Prep C_18_ column (5 mm, 20 mm×250 mm; Wako Chemicals USA, Inc., Richmond, VA USA) and a Sedere Sedex 75 Evaporative Light Scattering Detector (ELSD; SEDERE, Alfortville Cedex, France) for determination of relevant fractionation range. The ELSD drift tube was set at 50°C, and the air nebulization pressure at 3.5 bar. The dual solvent system used is a linear gradient program based on the method published by Mansour [86], with an extended run time, and substitutes (DCM) for chloroform. Starting with 98% acetonitrile (MeCN) and 2% DCM, the program ramps to 60% MeCN linearly by 100 minutes, holds at that ratio until 105 minutes, then ramps to 100% DCM by 110 minutes holding until 115 min, and returns to the starting conditions by 120 minutes. Methyl 12-methyltetradecanoate (C15∶0) and Methyl 16-methylheptadecanoate (C18∶0) branched FAMEs standards (Sigma) were used to determine the HPLC retention times of analytes of interest. Fractions were collected for each FAME species: 17∶1 eluted at 12.8 minutes, C 15∶0 eluted at 13.5, C17∶0 eluted at 16.6 minutes for subsequent GC-EI-MS and ^1^H-NMR analysis.

TLC purification of TAGs was performed as previously described for single solvent system [5] followed by scraping of the TAG species that were detected by water staining. Scraped TAGs were extracted with 1∶1 (vol/vol) chloroform methanol for 1 Hr prior to filtration with 0.2 µM PTFE membrane (VWR International). Extracts were dried under nitrogen then subjected to transesterification and analyzed by (GC-FID) [5].

### TAGs Cycle Comparative Analysis

*R. opacus* PD630 TAGs cycle genes were identified through metabolic reconstruction and literature-based curation. The genes assigned to biochemical reactions within the TAGs cycle were analyzed to identify all significant gene pairs (BLASTP, E< = 1e-5) followed by Markov clustering as implemented by TribeMCL [42]. Candidate gene families were then aligned using MAFFT [77] and manually curated in Jalview 2.6.1 [79] using neighbor joining trees and curation of alignments. Because we were classifying by EC number which is a broad classification of function and not necessarily homology, a strict cutoff for percent overlap or percent similarity was not used to retain as many members of particular EC category as annotated in KEGG (http://www.genome.jp/kegg/). A full list of curated EC # related Gene families implicated at each biochemical step of the TAGs cycle are represented with 11 other bacterial species in Table S4. Multiexperiment viewer [87] was used to hierarchically cluster the species in the TAGs cycle and visualize in a heat map. Clustering was pearson correlated with average linkage. The threshold for color-saturation was set to 15 genes per reaction category.

### Phenotype Growth Assays on 190 Compounds

Chemical compounds capable of supporting growth as a sole source of carbon were identified using a tetrazolium-based growth assay developed by Biolog Incorporated; wherein growth of cells and aerobic respiration were measured by Dye D (Biolog, Inc) reduction resulting in purple color and turbidity read at 590 nm absorbance in Fluorostar plate reader. Innoculation cultures were grown for 1 day on LB agar plates at 30°C from which 3.8E^−3^ ODu/well were transferred from 0.5 ml water resuspensions after measurement in nanodrop (Thermo Scientific) at 600 nm. During growth, plates were wrapped in aluminum foil and measured at 44, 72, and 96 hours. The measured A_590_ values were normalized by first subtracting values from uninoculated plates. The average of normalized values were background subtracted with average values of inoculated wells that lacked a carbon source (negative control). The background subtracted growth values were separated into chemical categories by filtering compound classifications in Excel. The normalized growth values for each compound were clustered using Cluster 3.0 [88] with centered correlation. Heat maps were generated in Java TreeView 1.1.3 [88]. Growth substrates were identified at 96 hours incubation for compounds that had A_590_ values >0.2.

## Supporting Information

Dataset S1 Multiple sequence alignment of *Rhodococcus* FAS type 1a protein. To visualize multiple sequence alignments of FAS type 1a protein 1) download jalview http://www.jalview.org/ 2) open Jalview 3) input alignment file: FAS_type1a_MSA.fa (Dataset S1) 4) load features features/annotations FAS_protein_domains_jalview.txt (Dataset S2).
(TXT)

Dataset S2 Protein domain annotation of FAS type 1a synthase. Load onto multiple sequence alignment according to instructions presented in Dataset S1.
(TXT)

Figure S1 Extensive synteny between three *Rhodococcus* species. Alignment of the assembled and stitched *R. opacus* PD630 draft genome with *R. jostii* RHA1 (top panel) and *R. opacus* B4 (bottom panel). Green lines indicate the end of completed genome replicons and purple lines show the ends of *R. opacus* PD630 assembled scaffolds that were aligned and orientated manually.
(EPS)

Figure S2 Outline of metabolic reconstructions steps used to generate the Opacuscyc14.5_working model.
(EPS)

Figure S3 Genomic and metabolic features of a comparative set of bacterial species. Left panel is a single-copy phylogenetic tree built from a common set of 701 genes present in all of the genomes of these select Actinomycetales. The 701 genes were compared at the DNA sequence level to establish phylogenetic relationships for each species. The bootstrap values are represented adjacent to the nodes. The branch length (scale bar) reflects the average number of substitutions/nucleotide. In the right panel, the predicted proteome from each organism was functionally categorized by protein homology according to KEGG orthology. The heat map represents hierarchical clustering of the number of genes in each category with the set of species being compared. Color scale bar on bottom indicates number of different genes in each category with a range from 0 to >400 genes. The bottom panel chart presents general genomic features of a comparative set of species.
(EPS)

Figure S4 Phylogenetic trees of acetyl- and propionoyl-CoA carboxylases. Closest matches of *R. opacus* PD630 proteins to AccA and AccD proteins that have been functionally characterized in *Mycobacterium* are designated with the number of each isozyme (AccA1,2,3 and AccD1,2,3,4,5,6). AccA proteins detected in each species represented as (species, number of homologous proteins): (*R. opacus* B4, 11), (*R. opacus* PD630, 12), (*R. jostii* RHA1, 11), (*M. vanbaalenii* PYR-1, 5), (*M. sp* JLS, 6), (*M. tuberculosis* H37Rv, 4), (*M. leprae* Br4923, 1). AccD proteins detected in each species represented as (species, # of homologous proteins): (*R. opacus* B4, 20), (*R. opacus* PD630, 21), (*R. jostii* RHA1, 21), (*M. vanbaalenii* PYR-1, 12), (*M. sp* JLS, 13), (*M. tuberculosis* H37Rv, 9), (*M. leprae* Br4923, 4).
(EPS)

Figure S5 The biochemical reactions of the TAGs cycle. The TAG metabolic cycle consists of 5 phases: 1) initiation to make acyl primers, 2) elongation by FAS to make C_16_–C_26_ fatty acyl-CoAs, 3) transesterification of fatty acyl-CoAs with glycerol, 4) hydrolysis of fatty acids from glycerol, and 5) β-oxidation of the released fatty acyl-CoAs. The EC numbers are used to describe the biochemical reactions and the color scheme, similar to Figure 3, indicates the number of genes implicated through metabolic reconstruction and TribeMCL gene-family analysis for each reaction.
(EPS)

Figure S6 A phylogenetic tree of the diacylglycerol acyl transferases (DGATs). The closest match in *R. opacus* PD630 to the genes defined previously in *R. jostii* RHA1 are labeled with an atf gene number outside of the circular phylogenetic-tree [30] (originally named through identification in *Acinetobacter baylyi*). DGAT/WS proteins detected in each species represented as (species, number of homologous proteins): (*R. opacus* B4, 9), (*R. opacus* PD630, 14), (*R. jostii* RHA1, 16), (*M. vanbaalenii* PYR-1, 12), (*M. sp* JLS, 14), (*M. tuberculosis* H37Rv, 12), (*M. leprae* Br4923, 1).
(EPS)

Figure S7 Glucose fermentation analytics for *R. opacus* PD630 and *R. jostii* RHA1. Top panel plots shows decreases in ammonium sulfate in the culture media (blue) and increases in residual cell dry weight (red) (total cell weight – total lipids) during fermentation on glucose. Bottom panel plots show depletion of media glucose concentration and increasing total fatty acids (green).
(EPS)

Figure S8 GC and EI coupled mass spectra of pentadecanoic acid. GC-MS of C_15∶0_ purified from *Rhodococcus* (GC-MS top panel, GC-EI-MS bottom panel).
(EPS)

Figure S9 GC and EI coupled mass spectra of heptadecanoic acid. GC-MS of C_17∶0_ purified from *Rhodococcus* (GC-MS top panel, GC-EI-MS bottom panel).
(EPS)

Figure S10 GC and EI coupled mass spectra of heptadecenoic acid. GC-MS of C_17∶1_ purified from *Rhodococcus* (GC-MS top panel, GC-EI-MS bottom panel).
(EPS)

Figure S11 ^1^H-NMR of FAMEs of C_15∶0_, C_17∶0_, and cis-C_17∶1_ purified from *Rhodococcus*. Control lipids were FAMEs of branched 16-methyl-heptadecanoate and linear pentadecanoate.
(EPS)

Figure S12 Screens of four bacterial species for growth on nitrogenous compounds. Nitrogen-containing compounds were clustered according to how *R. opacus* PD630, *R. jostii* RHA1, *C. glutamicum* 13032, and *R. eutropha* H16 were able to grow on them from 2–4 days. Yellow indicates evidence of growth. 38 Nitrogenous compounds including many amino acids were tested for growth. *R. opacus* PD630 catabolized 25 nitrogenous compounds as the sole carbon source (66%), *R. jostii* RHA1 catabolized 28 compounds (74%), *C. glutamicum* 8 (23%), and *R. eutropha* H16 11 (38%). The cyclical amino acid L-pyroglutamic acid was able to support growth in all species tested whereas the zwitterion L-glutamic acid was a weaker growth substrate. A notable feature of amino acid catabolism was observed for both *Rhodococcus* species and *R. eutropha* H16 contained the catabolic pathways for the branched amino acids, L-isoleucine, L-threonine, and L-valine catabolism. The catabolic pathways for branched amino acids result in the formation of propionoyl-CoA a substrate in the biosynthesis of stored odd-carbon lipids containing as presented here.
(EPS)

Figure S13 ORFs from genome annotation were searched for recognizable Pfam protein domains. The number of protein domains found within each species genomes are presented in a heat map with hierarchical-clustering clustering of species and rank-order of the most abundant domains in *R. opacus* PD630 (top to bottom).
(EPS)

Figure S14 The cholesterol degradation region of the *R. opacus* PD630 genome. The chromosomal region of *R. opacus PD630* genome containing 6 clusters of cholesterol degradation genes were mapped from the *R. jostii* RHA1 genome [61]. At the top of each cluster there is a nucleotide ruler for the stitched *R. opacus* PD630 genome. Below that are the genes with name and an orientation. Below the genes is a contig marker in blue that shows one contig without gaps contains the cholesterol degradation region for all six gene clusters. Below the contig marker there are multiple sequence alignments showing blocks of sequence homology in green (top strand) and purple (bottom strand). The names of the species containing the homology sequence are shown within the block of homology.
(EPS)

Table S1 Correlation table for growth of *R. opacus* PD630 on 190 chemical compounds between predictions from metabolic reconstruction and observed phenotypes. Correlation categories are named with respect to predictions from the metabolic reconstruction model of R. opacus PD630. A true positive encodes a complete compound degradation pathway in the metabolic reconstruction and the chemical compound supports growth of *R. opacus* PD630. A true negative lacks a complete compound degradation pathway and growth is not observed on that compound. A false negative lacks a complete degradation pathway but growth on the compound was observed. A false positive predicted growth but no growth was observed. The calculations and values for precison, recall, false-positive-rate, false-negative rate, specificity, and accuracy are detailed within the figure. The initial comparicyc_model metrics were compared to the more advanced opacus_working model metabolic reconstruction by calculating a delta-by-refinement; wherein the opacus_comparicyc values were subtracted from the opacus_working values.
(PDF)

Table S2 Four species of bacteria were screened for growth on 190 chemical compounds. Worksheet 1 compound normalized and background subtracted growth values. Column A) compounds used as a sole source of carbon. Column B) Chemical Abstract Service (CAS) chemical identification number for compounds tested as growth substrates. Column C) the chemical category for the compound tested. Column D) the average values after subtraction of the chemicals in the wells without cells 44 hours after inoculation of *R. opacus* PD630. Column E) the standard deviation of the compound subtracted values after 44 hours of growth. Column F) background subtracted growth wherein the average values of six negative controls that contain inoculated cells but no carbon growth substrate were used to further subtract 590 nm absorbance that is independent of growth on each carbon source. Columns (G–I) are processed in the same way for the 72 hour time point as was done for the 44 hour time points (Columns D–F). The 96 hour time points are represented in columns (J–L). *R. jostii* RHA1 growth value measurement for time points 44, 72, and 96 hours are presented in Columns (O–U). *C. glutamicum* 13032 growth value measurement for time points 44, 72, and 96 hour time points are presented in (Columns V-AD). *R. eutropha* H16 growth value measurement for time points 44, 72, and 96 hour time points are presented in Columns (AE–AM). Worksheet 2 presents the values from worksheet 1 ranked for *R. opacus* PD630 background subtracted growth values at the 96 hour time point. Worksheet 3 presents the values from worksheet 1 ranked for *R. jostii* RHA1 background subtracted growth values at the 96 hour time point. Worksheet 4 presents the values from worksheet 1 ranked for *C. glutamicum* 13032 background subtracted growth values at the 96 hour time point. Worksheet 5 presents the values from worksheet 1 ranked for *R. eutropha* H16 background subtracted growth values at the 96 hour time point.
(XLS)

Table S3 Genebank accession numbers for species used in comparative analysis. Column1_ Species name Column 2) Genbank genome accession numbers for comparative set of species.
(XLS)

Table S4 TribeMCL analysis of TAGs cycle genes implicated through metabolic reconstruction. Column A) presents the TribeMCL cluster identification number containing all homologous genes for 12 species. Column B) describes the bacterial species. Column C) presents the gene count for the whole TribeMCL analysis of the TAGs cycle. Column D) the EC number for the biochemical reactions within the TAGs cycle. Column E) LocusId is the gene identification (Broad) and locustag (NCBI). Column F) gene length. Column G) gene name.
(TXT)

## References

[1] McLeod MP, Warren RL, Hsiao WW, Araki N, Myhre M (2006). The complete genome of *Rhodococcus sp.* RHA1 provides insights into a catabolic powerhouse.. Proc Natl Acad Sci U S A.

[2] Alvarez HM, Mayer F, Fabritius D, Steinbuchel A (1996). Formation of intracytoplasmic lipid inclusions by *Rhodococcus opacus* strain PD630.. Arch Microbiol.

[3] Alvarez HM, Steinbuchel A (2002). Triacylglycerols in prokaryotic microorganisms.. Appl Microbiol Biotechnol.

[4] Hernandez M, Mohn W, Martinez E, Rost E, Alvarez A (2008). Biosynthesis of storage compounds by *Rhodococcus jostii* RHA1 and global identification of genes involved in their metabolism.. BMC genomics.

[5] Kurosawa K, Boccazzi P, de Almeida NM, Sinskey AJ (2010). High-cell-density batch fermentation of *Rhodococcus opacus* PD630 using a high glucose concentration for triacylglycerol production.. Journal of Biotechnology.

[6] Hughes J, Armitage YC, Symes KC (1998). Application of whole cell rhodococcal biocatalysts in acrylic polymer manufacture.. Antonie van Leeuwenhoek.

[7] Seto M, Kimbara K, Shimura M, Hatta T, Fukuda M (1995). A Novel Transformation of Polychlorinated Biphenyls by *Rhodococcus sp*. Strain RHA1.. Appl Environ Microbiol.

[8] Robrock KR, Mohn WW, Eltis LD, Alvarez-Cohen L (2010). Biphenyl and ethylbenzene dioxygenases of *Rhodococcus jostii* RHA1 transform PBDEs.. Biotechnol Bioeng.

[9] Goncalves ER, Hara H, Miyazawa D, Davies JE, Eltis LD (2006). Transcriptomic assessment of isozymes in the biphenyl pathway of *Rhodococcus* sp. strain RHA1.. Appl Environ Microbiol.

[10] Mathieu JM, Mohn WW, Eltis LD, LeBlanc JC, Stewart GR (2010). 7-ketocholesterol catabolism by *Rhodococcus jostii* RHA1.. Appl Environ Microbiol.

[11] Puglisi E, Cahill MJ, Lessard PA, Capri E, Sinskey AJ (2010). Transcriptional response of *Rhodococcus aetherivorans* I24 to polychlorinated biphenyl-contaminated sediments.. Microb Ecol.

[12] Peoples OP, Sinskey AJ (1989). Poly-beta-hydroxybutyrate (PHB) biosynthesis in *Alcaligenes eutrophus* H16. Identification and characterization of the PHB polymerase gene (phbC).. Journal of Biological Chemistry.

[13] Slater SC, Voige WH, Dennis DE (1988). Cloning and expression in Escherichia coli of the *Alcaligenes eutrophus* H16 poly-beta-hydroxybutyrate biosynthetic pathway.. Journal of Bacteriology.

[14] Jendrossek D (2009). Polyhydroxyalkanoate Granules Are Complex Subcellular Organelles (Carbonosomes).. J Bacteriol.

[15] Rajakumari S, Grillitsch K, Daum G (2008). Synthesis and turnover of non-polar lipids in yeast.. Progress in Lipid Research.

[16] Alvarez HM, A S (2010). Biology of *Rhodococcus*.. Microbiology Monographs.

[17] Argyrou A, Vetting M, Blanchard J (2007). New insight into the mechanism of action of and resistance to isoniazid: interaction of *Mycobacterium tuberculosis* enoyl-ACP reductase with INH-NADP.. Journal of the American Chemical Society.

[18] Timmins G, Deretic V (2006). Mechanisms of action of isoniazid.. Molecular Microbiology.

[19] Raman K, Rajagopalan P, Chandra N (2005). Flux Balance Analysis of Mycolic Acid Pathway: Targets for Anti-Tubercular Drugs.. PLoS Comput Biol.

[20] Zimhony O, Cox J, Welch J, Vilcheze C, Jacobs W (2000). Pyrazinamide inhibits the eukaryotic-like fatty acid synthetase I (FASI) of *Mycobacterium tuberculosis*.. Nature Medicine.

[21] Schweizer E, Hofmann J (2004). Microbial type I fatty acid synthases (FAS): major players in a network of cellular FAS systems.. Microbiology and molecular biology reviews: MMBR.

[22] Sutcliffe IC (1998). Cell envelope composition and organisation in the genus *Rhodococcus*.. Antonie Van Leeuwenhoek.

[23] Hsu F-F, Soehl K, Turk J, Haas A (2011). Characterization of mycolic acids from the pathogen *Rhodococcus equi* by tandem mass spectrometry with electrospray ionization.. Analytical Biochemistry.

[24] Karp PD, Paley SM, Krummenacker M, Latendresse M, Dale JM (2010). Pathway Tools version 13.0: integrated software for pathway/genome informatics and systems biology.. Brief Bioinform.

[25] Arakaki A, Huang Y, Skolnick J (2009). EFICAz2: enzyme function inference by a combined approach enhanced by machine learning.. BMC Bioinformatics.

[26] Kikuchi S, Rainwater DL, Kolattukudy PE (1992). Purification and characterization of an unusually large fatty acid synthase from *Mycobacterium tuberculosis* var. bovis BCG.. Archives of Biochemistry and Biophysics.

[27] Vance DE, Mitsuhashi O, Bloch K (1973). Purification and properties of the fatty acid synthetase from *Mycobacterium phlei*.. J Biol Chem.

[28] Wu M, Eisen JA (2008). A simple, fast, and accurate method of phylogenomic inference.. Genome Biol.

[29] Kalscheuer R, Steinbuchel A (2003). A novel bifunctional wax ester synthase/acyl-CoA:diacylglycerol acyltransferase mediates wax ester and triacylglycerol biosynthesis in *Acinetobacter calcoaceticus* ADP1.. J Biol Chem.

[30] Alvarez AF, Alvarez HM, Kalscheuer R, Waltermann M, Steinbuchel A (2008). Cloning and characterization of a gene involved in triacylglycerol biosynthesis and identification of additional homologous genes in the oleaginous bacterium *Rhodococcus opacus* PD630.. Microbiology.

[31] Gande R, Dover LG, Krumbach K, Besra GS, Sahm H (2007). The two carboxylases of *Corynebacterium glutamicum* essential for fatty acid and mycolic acid synthesis.. J Bacteriol.

[32] Portevin D, de Sousa-D'Auria C, Montrozier H, Houssin C, Stella A (2005). The acyl-AMP ligase FadD32 and AccD4-containing acyl-CoA carboxylase are required for the synthesis of mycolic acids and essential for mycobacterial growth: identification of the carboxylation product and determination of the acyl-CoA carboxylase components.. J Biol Chem.

[33] Gande R, Gibson KJ, Brown AK, Krumbach K, Dover LG (2004). Acyl-CoA carboxylases (accD2 and accD3), together with a unique polyketide synthase (Cg-pks), are key to mycolic acid biosynthesis in Corynebacterianeae such as *Corynebacterium glutamicum* and *Mycobacterium tuberculosis*.. J Biol Chem.

[34] Diacovich L, Peiru S, Kurth D, Rodriguez E, Podesta F (2002). Kinetic and Structural Analysis of a New Group of Acyl-CoA Carboxylases Found in *Streptomyces coelicolor* A3(2).. J Biol Chem.

[35] Rainwater DL, Kolattukudy PE (1985). Fatty acid biosynthesis in *Mycobacterium tuberculosis* var. bovis Bacillus Calmette-Guerin. Purification and characterization of a novel fatty acid synthase, mycocerosic acid synthase, which elongates n-fatty acyl-CoA with methylmalonyl-CoA.. Journal of Biological Chemistry.

[36] Matsunaga I, Bhatt A, Young DC, Cheng TY, Eyles SJ (2004). *Mycobacterium tuberculosis* pks12 produces a novel polyketide presented by CD1c to T cells.. J Exp Med.

[37] Sirakova T, Dubey V, Kim H-J, Cynamon M, Kolattukudy P (2003). The Largest Open Reading Frame (pks12) in the *Mycobacterium tuberculosis* Genome Is Involved in Pathogenesis and Dimycocerosyl Phthiocerol Synthesis.. Infect Immun.

[38] Sirakova TD, Dubey VS, Cynamon MH, Kolattukudy PE (2003). Attenuation of *Mycobacterium tuberculosis* by disruption of a mas-like gene or a chalcone synthase-like gene, which causes deficiency in dimycocerosyl phthiocerol synthesis.. J Bacteriol.

[39] Sirakova TD, Thirumala AK, Dubey VS, Sprecher H, Kolattukudy PE (2001). The Mycobacterium tuberculosis pks2 gene encodes the synthase for the hepta- and octamethyl-branched fatty acids required for sulfolipid synthesis.. The Journal of Biological Chemistry.

[40] Grafe U, Reinhardt G, Krebs D, Roth M, Bormann EJ (1982). Biochemical characteristics of non-streptomycin-producing mutants of *Streptomyces griseus*. II. Lipids and fatty acid composition of vegetative mycelia.. Z Allg Mikrobiol.

[41] Kaddor C, Biermann K, Kalscheuer R, Steinbuchel A (2009). Analysis of neutral lipid biosynthesis in *Streptomyces avermitilis* MA-4680 and characterization of an acyltransferase involved herein.. Appl Microbiol Biotechnol.

[42] Enright AJ, Kunin V, Ouzounis CA (2003). Protein families and TRIBES in genome sequence space.. Nucleic Acids Res.

[43] Chopra T, Banerjee S, Gupta S, Yadav G, Anand S (2008). Novel Intermolecular Iterative Mechanism for Biosynthesis of Mycoketide Catalyzed by a Bimodular Polyketide Synthase.. PLoS Biol.

[44] Rainwater DL, Kolattukudy PE (1983). Synthesis of mycocerosic acids from methylmalonyl coenzyme A by cell-free extracts of *Mycobacterium tuberculosis* var. bovis BCG.. Journal of Biological Chemistry.

[45] Horswill AR, Escalante-Semerena JC (1999). Salmonella typhimurium LT2 catabolizes propionate via the 2-methylcitric acid cycle.. J Bacteriol.

[46] David F, Tienpont B, Sandra P (2008). Chemotaxonomy of bacteria by comprehensive GC and GC-MS in electron impact and chemical ionisation mode.. Journal of Separation Science.

[47] Lenz O, Ludwig M, Schubert T, Burstel I, Ganskow S (2010). H2 conversion in the presence of O2 as performed by the membrane-bound [NiFe]-hydrogenase of *Ralstonia eutropha*.. Chemphyschem.

[48] Pohlmann A, Fricke WF, Reinecke F, Kusian B, Liesegang H (2006). Genome sequence of the bioplastic-producing “Knallgas” bacterium *Ralstonia eutropha* H16.. Nat Biotechnol.

[49] Schwartz E, Voigt B, Zuhlke D, Pohlmann A, Lenz O (2009). A proteomic view of the facultatively chemolithoautotrophic lifestyle of *Ralstonia eutropha* H16.. Proteomics.

[50] Parker SK, Curtin KM, Vasil ML (2007). Purification and characterization of mycobacterial phospholipase A: an activity associated with mycobacterial cutinase.. J Bacteriol.

[51] Vimr E, Kalivoda K, Deszo E, Steenbergen S (2004). Diversity of microbial sialic acid metabolism.. Microbiology and molecular biology reviews: MMBR.

[52] Severi E, Hood DW, Thomas GH (2007). Sialic acid utilization by bacterial pathogens.. Microbiology.

[53] Post D, Mungur R, Gibson B, Munson R (2005). Identification of a novel sialic acid transporter in *Haemophilus ducreyi*.. Infection and Immunity.

[54] Severi E, Hosie A, Hawkhead J, Thomas G (2009). Characterization of a novel sialic acid transporter of the sodium solute symporter (SSS) family and *in vivo* comparison with known bacterial sialic acid transporters.. FEMS Microbiology Letters.

[55] Hunter RL, Olsen M, Jagannath C, Actor JK (2006). Trehalose 6,6′-dimycolate and lipid in the pathogenesis of caseating granulomas of tuberculosis in mice.. Am J Pathol.

[56] Rengarajan J, Bloom BR, Rubin EJ (2005). Genome-wide requirements for *Mycobacterium tuberculosis* adaptation and survival in macrophages.. Proc Natl Acad Sci U S A.

[57] Pandey AK, Sassetti CM (2008). Mycobacterial persistence requires the utilization of host cholesterol.. Proc Natl Acad Sci U S A.

[58] Hu Y, van der Geize R, Besra GS, Gurcha SS, Liu A (2010). 3-Ketosteroid 9alpha-hydroxylase is an essential factor in the pathogenesis of *Mycobacterium tuberculosis*.. Mol Microbiol.

[59] Yang X, Nesbitt NM, Dubnau E, Smith I, Sampson NS (2009). Cholesterol metabolism increases the metabolic pool of propionate in *Mycobacterium tuberculosis*.. Biochemistry.

[60] Van der Geize R, Yam K, Heuser T, Wilbrink MH, Hara H (2007). A gene cluster encoding cholesterol catabolism in a soil actinomycete provides insight into *Mycobacterium tuberculosis* survival in macrophages.. Proceedings of the National Academy of Sciences.

[61] Yam KC, Okamoto S, Roberts JN, Eltis LD (2011). Adventures in *Rhodococcus* - from steroids to explosives.. Can J Microbiol.

[62] Lomakin IB, Xiong Y, Steitz TA (2007). The Crystal Structure of Yeast Fatty Acid Synthase, a Cellular Machine with Eight Active Sites Working Together.. Cell.

[63] MacEachran DP, Prophete ME, Sinskey AJ (2010). The *Rhodococcus opacus* PD630 heparin-binding hemagglutinin homolog TadA mediates lipid body formation.. Appl Environ Microbiol.

[64] Lessard PA, O'Brien XM, Currie DH, Sinskey AJ (2004). pB264, a small, mobilizable, temperature sensitive plasmid from *Rhodococcus*.. BMC Microbiol.

[65] Isono K, McIninch JD, Borodovsky M (1994). Characteristic features of the nucleotide sequences of yeast mitochondrial ribosomal protein genes as analyzed by computer program GeneMark.. DNA Res.

[66] Delcher AL, Harmon D, Kasif S, White O, Salzberg SL (1999). Improved microbial gene identification with GLIMMER.. Nucleic Acids Res.

[67] Delcher AL, Phillippy A, Carlton J, Salzberg SL (2002). Fast algorithms for large-scale genome alignment and comparison.. Nucleic Acids Res.

[68] Brudno M, Do CB, Cooper GM, Kim MF, Davydov E (2003). LAGAN and Multi-LAGAN: efficient tools for large-scale multiple alignment of genomic DNA.. Genome Res.

[69] Finn RD, Mistry J, Schuster-Bockler B, Griffiths-Jones S, Hollich V (2006). Pfam: clans, web tools and services.. Nucleic Acids Res.

[70] Lagesen K, Hallin P, Rodland EA, Staerfeldt HH, Rognes T (2007). RNAmmer: consistent and rapid annotation of ribosomal RNA genes.. Nucleic Acids Res.

[71] Lowe TM, Eddy SR (1997). tRNAscan-SE: a program for improved detection of transfer RNA genes in genomic sequence.. Nucleic Acids Res.

[72] Griffiths-Jones S, Moxon S, Marshall M, Khanna A, Eddy SR (2005). Rfam: annotating non-coding RNAs in complete genomes.. Nucleic Acids Res.

[73] Romero PR, Karp PD (2004). Using functional and organizational information to improve genome-wide computational prediction of transcription units on pathway-genome databases.. Bioinformatics.

[74] Lee T, Paulsen I, Karp P (2008). Annotation-based inference of transporter function.. Bioinformatics.

[75] Green M, Karp P (2004). A Bayesian method for identifying missing enzymes in predicted metabolic pathway databases.. BMC Bioinformatics.

[76] Li L, Stoeckert C, Roos D (2003). OrthoMCL: Identification of Ortholog Groups for Eukaryotic Genomes.. Genome Research.

[77] Katoh K, Kuma K, Toh H, Miyata T (2005). MAFFT version 5: improvement in accuracy of multiple sequence alignment.. Nucleic Acids Res.

[78] Stamatakis A (2006). RAxML-VI-HPC: maximum likelihood-based phylogenetic analyses with thousands of taxa and mixed models.. Bioinformatics.

[79] Waterhouse AM, Procter JB, Martin DM, Clamp M, Barton GJ (2009). Jalview Version 2–a multiple sequence alignment editor and analysis workbench.. Bioinformatics.

[80] Marchler-Bauer A, Lu S, Anderson JB, Chitsaz F, Derbyshire MK (2011). CDD: a Conserved Domain Database for the functional annotation of proteins.. Nucleic Acids Res.

[81] Edgar R (2004). MUSCLE: multiple sequence alignment with high accuracy and high throughput.. Nucleic acids research.

[82] Larkin MA, Blackshields G, Brown NP, Chenna R, McGettigan PA (2007). Clustal W and Clustal X version 2.0.. Bioinformatics.

[83] Abascal F, Zardoya R, Posada D (2005). ProtTest: selection of best-fit models of protein evolution.. Bioinformatics.

[84] Guindon S, Gascuel O (2003). A simple, fast, and accurate algorithm to estimate large phylogenies by maximum likelihood.. Systematic biology.

[85] Letunic I, Bork P (2007). Interactive Tree Of Life (iTOL): an online tool for phylogenetic tree display and annotation.. Bioinformatics.

[86] Mansour MP (2005). Reversed-phase high-performance liquid chromatography purification of methyl esters of C(16)–C(28) polyunsaturated fatty acids in microalgae, including octacosaoctaenoic acid [28:8(n-3)].. J Chromatogr A.

[87] Saeed AI, Bhagabati NK, Braisted JC, Liang W, Sharov V (2006). TM4 microarray software suite.. Methods Enzymol.

[88] de Hoon MJ, Imoto S, Nolan J, Miyano S (2004). Open source clustering software.. Bioinformatics.

