# Supplementary material for: Comparative and Functional Genomics of Rhodococcus opacus PD630 for Biofuels Development
Source: PLoS Genet. 2011 Sep 8;7(9):e1002219. doi: 10.1371/journal.pgen.1002219 (PMC3169528; doi:10.1371/journal.pgen.1002219)
Supplement: Table S1 — Correlation table for growth of R. opacus PD630 on 190 chemical compounds between predictions from metabolic reconstruction and observed phenotypes. Correlation categories are named with respect to predictions from the metabolic reconstruction model of R. opacus PD630. A true positive encodes a complete compound degradation pathway in the metabolic reconstruction and the chemical compound supports growth of R. opacus PD630. A true negative lacks a complete compound degradation pathway and growth is not observed on that compound. A false negative lacks a complete degradation pathway but growth on the compound was observed. A false positive predicted growth but no growth was observed. The calculations and values for precison, recall, false-positive-rate, false-negative rate, specificity, and accuracy are detailed within the figure. The initial comparicyc_model metrics were compared to the more advanced opacus_working model metabolic reconstruction by calculating a delta-by-refinement; wherein the opacus_comparicyc values were subtracted from the opacus_working values. (PDF) [file pgen.1002219.s017.pdf]

Table 1. Phenotypic Correlations with Growth Predictions from Metabolic Reconstruction

True Positives (metabolic reconstruction predicted **growth** and **growth** was observed for each compound)

True Negatives (metabolic reconstruction predicted **no growth** and **no growth** was observed for each compound)

False Negatives (metabolic reconstruction predicted **no growth** but **growth** was observed for each compound)

False Positives (metabolic reconstruction predicted **growth** but **no growth** was observed for each compound)

Predicted vs observed categories	opacus_compare	opacus_working	delta-by-refinement
true positives:	39.00	49.00	10.00
false positives:	21.00	20.00	-1.00
true negatives:	80.00	81.00	1.00
false negatives:	54.00	44.00	-10.00
precision: TP/(TP + FP)	0.65	0.71	0.06
recall: TP/(TP + FN)	0.42	0.53	0.11
false-positive-rate: FP/(FP + TN)	0.21	0.20	-0.01
false-negative-rate: FN/(TP + FN)	0.58	0.47	-0.11
specificity: TN/(TN + FP)	0.79	0.80	0.01
accuracy: (TP + TN)/(TP + FP + TN + FN)	0.61	0.67	0.06
